# Application of chloroplast genome in the identification of *Phyllanthus urinaria* and its common adulterants

**DOI:** 10.3389/fpls.2022.1099856

**Published:** 2023-01-06

**Authors:** Hui Fang, Guona Dai, Binbin Liao, Ping Zhou, Yinglin Liu

**Affiliations:** College of Pharmaceutical Science, Dali University, Dali, China

**Keywords:** *Phyllanthus urinaria*, chloroplast genome, species identification, molecular marker, phylogenetic

## Abstract

**Background:**

*Phyllanthus urinaria* L. is extensively used as ethnopharmacological material in China. In the local marketplace, this medicine can be accidentally contaminated, deliberately substituted, or mixed with other related species. The contaminants in herbal products are a threat to consumer safety. Due to the scarcity of genetic information on *Phyllanthus* plants, more molecular markers are needed to avoid misidentification.

**Methods:**

In this study, the complete chloroplast genome of nine species of the genus *Phyllanthus* was *de novo* assembled and characterized.

**Results:**

This study revealed that all of these species exhibited a conserved quadripartite structure, which includes a large single copy (LSC) region and small single copy (SSC) region, and two copies of inverted repeat regions (IRa and IRb), which separate the LSC and SSC regions. And the genome structure, codon usage, and repeat sequences were highly conserved and showed similarities among the nine species. Three highly variable regions (*trnS-GCU-trnG-UCC*, *trnT-UGU-trnL-UAA*, and *petA-psbJ*) might be helpful as potential molecular markers for identifying P. urinaria and its contaminants. In addition, the molecular clock analysis results showed that the divergence time of the genus *Phyllanthus* might occur at ~ 48.72 Ma.

**Conclusion:**

This study provides valuable information for further species identification, evolution, and phylogenetic research of *Phyllanthus*.

## 1 Introduction

*Phyllanthus urinaria* L. belongs to the family Euphorbiaceae, is listed in the dictionary of Chinese ethnic medicine, and the Chinese name is “Yexiazhu” (Guo et al., 2017). The herbal *P. urinaria* has crucial medicinal value in anti-diarrheal, anti-inflammatory, jaundice, diabetes, malaria, hepatitis B, and liver diseases (Chudapongse et al., 2010; Geethangili and Ding, 2018). The previous survey has revealed that *P. urinaria* is generally contaminated with other common adulterants, such as *P. acidus* (L.) Skeel, *P. amarus* Schumacher & Thonning, *P. reticulatus* Poir., *P. niruri* L., *P. emblica* L., *P. pulcher* Well. ex Muell. Arg., and *P. debilis* Klein ex Willd. (Manissorn et al., 2010; Srirama et al., 2010; Kiran et al., 2021). These adulterants are usually of poor quality, and some might even be toxic (Adedapo et al., 2005; Geng et al., 2021). As the morphology of these species are interchangeable, similar, and indistinguishable, the identification of these species remains controversial, which may impair their clinical safety and efficacy (Sarin et al., 2013; Kiran et al., 2021). Therefore, it is essential to develop a method for accurately identifying *P. urinaria* and its common contaminants.

With the rapid development of molecular technology, molecular identification has made significant progress in Chinese medicine, especially molecular markers, a technique that involves sequencing specific sections of the genome to identify differences between individuals of different species or populations (Xiong et al., 2018). Recent studies have revealed high levels of genetic diversity and a lack of phylogenetic resolution within species of *Phyllanthus* (Pruesapan et al., 2012; Bouman et al., 2021). Universal DNA barcodes, such as ITS, *psbA-trnH*, *trnL*, *psbK-psbI*, *rpoC1*, and *trnL-trnF*, have been used to identify *P. urinaria* and its adulterants (Manissorn et al., 2010; Srirama et al., 2010; Inglis et al., 2018; Kiran et al., 2021). However, some common adulterants were not included in these investigations, and there are inherent limitations to single-locus DNA barcodes (Heinze, 2007; Li et al., 2015). Therefore, more scientific and accurate identification methods must be developed. The chloroplast (cp) is an essential organelle that plays a crucial role in plant photosynthesis and several other critical biochemical processes (Neuhaus and Emes, 2000). Compared with the traditional DNA fragments, the cp genome was relatively conserved and slightly varied, which has been applied to many research fields, such as species identification and the development of molecular markers (Abdullah et al., 2020; Li et al., 2022; Wang et al., 2022). The method has been widely used for identifying *Paris*, *Polygonatum*, *Vicatia*, and their adulterants (Guan et al., 2022; Jiang et al., 2022; Wang et al., 2022). Recently, although the complete cp genomes of Phyllanthaceae species have been reported and the high-resolution phylogenetic tree was reconstructed (Rehman et al., 2021), the purpose of this study was to clarify the genome evolution in Phyllanthaceae and identify the polymorphic loci for phylogenetic inference. To our knowledge, no reports use cp genomes to compare *P. urinaria* with its common adulterants.

Our study aims to: (i) contribute new fully-sequenced cp genomes of *Phyllanthus* and improve the understanding of the overall structure of these genomes, (ii) perform comparative analyses and elucidate the phylogenetic evolution of the *Phyllanthus*, and (iii) screen potential molecular markers to distinguish *P. urinaria* from its contaminants. In the current work, the complete cp genomes of nine *Phyllanthus* species were sequenced, *de novo* assembled, and annotated. These genomes were then used in a comparative analysis of genome structure and evolution relationships. This research expands the genomic resources available for *Phyllanthus* and provides valuable information support for the phylogenetic analysis and identification of the *Phyllanthus*, as well as for the safe applications of *P. urinaria.*

## 2 Material and methods

### 2.1 Plant and DNA sources

The fresh and healthy leaves for nine species of *P. acidus*, *P. amarus*, *P. reticulatus*, *P. urinaria*, *P. niruri*, *P. niruri* subsp*. lathyroides*, *P. emblica*, *P. pulcher*, and *P. franchetianus* were collected from Dali and Xishuangbanna, Yunnan Province, China. The detailed information per sample is available in **Supplementary Table 1**. The samples were identified following the taxonomic key and external morphology diagnosis proposed by related literature (Webster and Carpenter, 2008). The voucher specimens were preserved at the herbarium of Dali University. The fresh leaf of nine species was frozen in liquid nitrogen and stored in a 4°C refrigerator for DNA extraction. Total DNA was extracted using a modified cetyl trimethyl ammonium bromide (CTAB) procedure (Allen et al., 2006). DNA quality and quantity were assessed using a NanoDrop spectrophotometer (ND-2000; Thermo Fisher Scientific, USA) and agarose gel electrophoresis.

### 2.2 DNA sequencing, assembly and annotation

Purified high-quality genomic DNA was broken into short fragments of approximately 350 bp, and paired-end (PE) libraries were constructed by adding A-tails, PCR amplification, and other steps, followed by sequencing in 150 bp paired-end mode on an Illumina NovaSeq 6000 platform. The high-quality reads were assembled using GetOrganelle v1.7.5 (Jin et al., 2020) and then annotated by cpGAVAS2 (http://47.96.249.172:16019/analyzer/annotate) and (GeSeq, RRID : SCR_017336) (https://chlorobox.mpimp-golm.mpg.de/geseq.html) (Tillich et al., 2017; Shi et al., 2019). The annotations of tRNA genes were confirmed by using (tRNAscan-SE v.2.03, RRID : SCR_010835) (http://lowelab.ucsc.edu/tRNAscan-SE/) (Schattner et al., 2005). Annotated cp genomes sequences were submitted to GenBank and are available under accession numbers OP009343-OP009351 (**Table 1**). Fully annotated cp genome circular diagrams were drawn by OrganellarGenomeDRAW (OGDRAW, RRID : SCR_017337) (https://chlorobox.mpimp-golm.mpg.de/OGDraw.html) (Greiner et al., 2019).

**Table 1:** Cp genomes features of nine species of *Phyllanthus*.

Genome features	*P. acidus*	*P. amarus*	*P. reticulatus*	*P. urinaria*	*P. niruri*	*P. niruri* subsp. *lathyroides*	*P. emblica*	*P. pulcher*	*P. franchetianus*
**Genome size (bp)**	156,331	155,790	156,610	153,850	155,900	143,563	155,841	155,589	155,598
**LSC size (bp)**	85,807	85,185	85,868	83,714	85,307	91,305	85,721	85,533	85,533
**SSC size (bp)**	19,262	19,015	19,182	18,780	19,015	18,986	18,950	18790	18,799
**IRa/IRb size (bp)**	25,631	25,795	25,780	25,678	25,789	16,771	25,585	25,633	25,633
**Total GC content (%)**	36.9	36.6	36.6	36.9	36.6	36.8	36.8	36.8	36.8
**GC content in LSC (%)**	34.6	34.3	34.3	34.5	34.2	34.9	34.5	34.4	34.4
**GC content in S.S.C. (%)**	30.6	30.0	30.2	30.6	30.0	30.1	30.4	30.9	30.9
**GC content in IRa/IRb (%)**	43.1	42.9	42.9	43.0	42.9	45.6	43.1	42.9	42.9
**Number of genes**	126	125	125	122	125	118	126	123	123
**Protein-coding genes**	82	81	82	79	81	75	82	79	79
**tRNA genes**	36	36	35	35	36	35	36	36	36
**rRNA genes**	8	8	8	8	8	8	8	8	8
**Accession numbers in GenBank**	OP009343	OP009344	OP009345	OP009346	OP009347	OP009348	OP009349	OP009350	OP009351

### 2.3 Genome structure and comparisons analysis

Forward (F), palindromic (P), reverse (R), and complementary (C) were identified using the REPuter (https://bibiserv.cebitec.uni-bielefeld.de/reputer/) tool (Kurtz et al., 2001). The criteria for identifying repeats include a minimum repetition size of 30 bp and a 90% similarity between repeat pairs, calculated by assigning a value of 3 to the altered sequence. In addition, (MISA, RRID : SCR_010765) (http://pgrc.ipk-gatersleben.de/misa/) software was used to identify simple sequence repeats (SSRs) (Beier et al., 2017). We followed conventional standards for identifying chloroplast and mitochondrial SSRs, including a minimum stretch of 10 for mono-, 5 for di-, 4 for tri-, and 3 for tetra-, penta- and hexanucleotide repeats and a minimum distance of 100 bp between compound SSRs. Relative synonymous codon usage (RSCU) was analyzed by CodonW v.1.4.2 (Sharp et al., 1986). Also, Tbtools v1.098761 used a heatmap to show the values of RSCU (Chen et al., 2020).

For comparative analysis of genes, tRNA, repeat content, genome size, and GC content were assessed by (Geneious v.2022.2.1, RRID : SCR_010519) (http://www.geneious.com/) software (Kearse et al., 2012). The software mVISTA (https://genome.lbl.gov/vista/index.shtml) (Frazer et al., 2004) in Shuffle-LAGAN mode (Brudno et al., 2003) was used to compare the nine *de novo* cp genome sequences, *P. amarus* (GenBank OP009344) was used as the reference genome. IRscope (https://irscope.shinyapps.io/irapp/) was used to analyze inverted repeat region contraction and expansion at the junctions of cp genomes (Amiryousefi et al., 2018). The cp genomes were aligned in (MAFFT, RRID : SCR_011811) (https://mafft.cbrc.jp/alignment/server/). Additionally, the nucleotide variability (Pi) across the cp genome sequences was performed in (DnaSP v.6.12.03, RRID : SCR_003067) (http://www.ub.edu/dnasp/) (Rozas et al., 2017), with a window length of 600 bp and step size of 200 bp. A value of Pi higher than 0.05 was recommended as mutational hotspots (Ren et al., 2022).

### 2.4 Phylogenetic analysis and divergence times analysis

For the phylogenetic analysis, 38 Euphorbiaceae taxa initially consisted of 29 species downloaded from NCBI (**Table S2**) and the 9 species presented here (**Table 1**). At the same time, two species, *Daphniphyllum oldhamii* (GenBank NC037883) and *D. macropodum* (GenBank MN496060) were selected as outgroups (Chase et al., 2016). A total of 40 cp genomes were aligned using MAFFT with the default parameters and trimmed using (TrimAl v.1.4, RRID : SCR_017334) (http://trimal.cgenomics.org/) with an automated option (Katoh and Standley, 2013). The best-fit model of nucleotide substitution was selected using ModelFinder (Kalyaanamoorthy et al., 2017) with the Bayesian information criterion as implemented in (IQ-tree v.1.6.12, RRID : SCR_017254) (http://www.iqtree.org/) (Nguyen et al., 2015). The alignment was also evaluated using bootstrap analysis on 1,000 in a maximum likelihood (ML) by IQ-tree v.1.6.12, with the following parameters: iqtree -s input -m TVM+F+R3 -bb 1000 -alrt 1000 -nt AUTO -o NC_037883, MN_496060. Besides, the neighbor-joining (NJ) tree was constructed using (MEGA X v.10.2.6, RRID : SCR_000667) (http://megasoftware.net/), and the bootstrap testing was performed with 1,000 repetitions (Kumar et al., 2018).

For analysis of divergence times, the molecular clock tree was constructed based on an ML tree using MEGA X v.10.2.6 (Kumar et al., 2018; Mello, 2018). The relevant divergence times were executed in the (TimeTree, RRID : SCR_021162) (http://www.timetree.org/) Resource (Kumar et al., 2017). Four calibration points were used to restrict each node: (F1) 110.9–121.0 Ma for the root node, (F2) 48.6–55.8 Ma for Phyllanthoideae stem age, (F3) 3.5–74.3 Ma for Acalyphoideae crown age, and (F4) 21.4–89 Ma for Euphorbioideae + Crotonoideae.

## 3 Results

### 3.1 Genome structure

**Figure 1:**
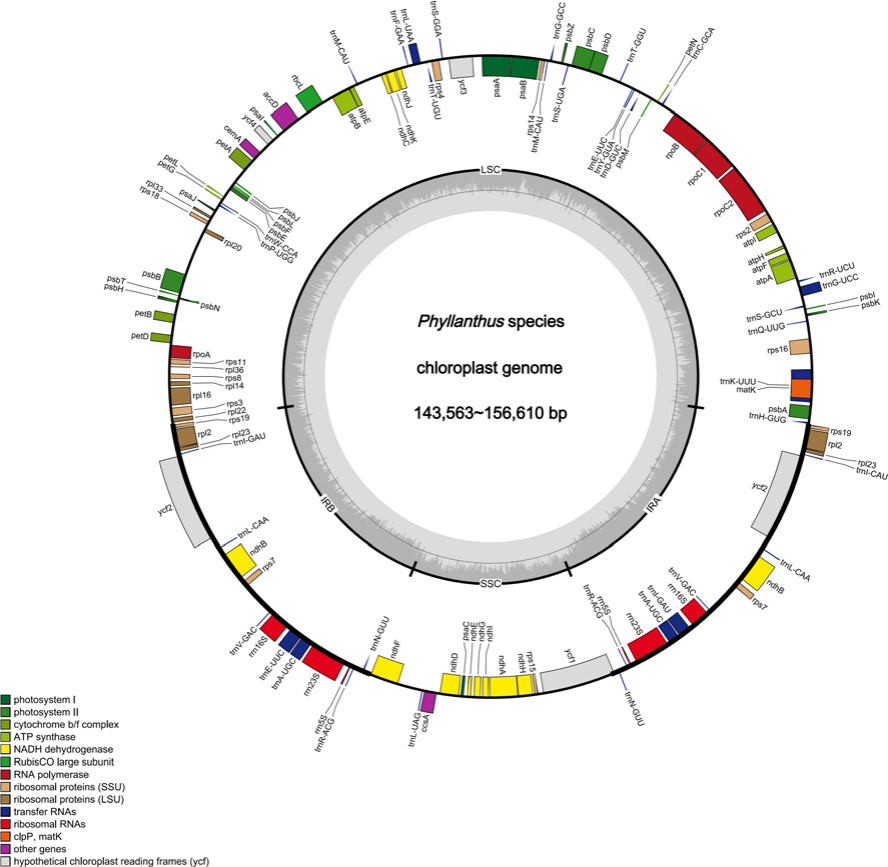
Cp genomes map of *Phyllanthus*. Genes inside and outside the circle are transcribed clockwise and counter-clockwise.

The raw data of nine species were filtered to remove adapters and low-quality reads. Approximately 2.24–4.09 Gb data were obtained for each species. The cp genomes of these nine species are small circular DNA molecules with sizes in the range of 143,563 bp (*P. niruri* subsp. *lathyroides*) to 156,610 bp (*P. reticulatus*) (**Figure 1**), with the typical quadripartite structure of land plant cp genomes consisting of two inverted repeats (IRa and IRb) separated by large single copy (LSC) and small single copy (SSC) regions, respectively. The size of LSC ranged from 83,714 bp (*P. urinaria*) to 91,305 bp (*P. niruri* subsp. *lathyroides*), SSC ranged from 18,780 bp (*P. urinaria*) to 19,262 bp (*P. acidus*), and the size of each IR region ranged from 16,771 bp (*P. niruri* subsp. *lathyroides*) to 25,795 bp (*P. amarus*). Moreover, the GC content in the IR region (42.9%–45.6%) was higher than LSC (34.2%–34.9%) and SSC (30.0%–30.9%) (**Table 1**).

In addition, a total of 118–126 genes were identified, which comprised 75–82 protein-coding genes, 35–36 tRNAs, and 8 rRNAs (**Table 1**), whereas the number of genes varies in species due to IRs contraction and expansion. These genes were divided into three parts, of which 45 genes belong to photosynthesis-related genes (photosystem I, photosystem II, NADPH dehydrogenase, cytochrome b/f complex ATP synthase, and rubisco), 27 genes belong to self-replication (the large subunit of the ribosome, small subunit of the ribosome, and DNA dependent RNA polymerase), and the remaining genes belong to other genes (acetyl-CoA-carboxylase, c-type cytochrome synthesis genes, envelop membrane proteins, proteases, and maturase) (**Table S3**). Moreover, 17 genes each contained one intron, among them *rpl2* (×2), *ndhB* (×2), *trnI*-*GAU* (×2), and *trnA*-*UGC* (×2), which were located in the IR, and the genes (*trnK-UUU*, *rps16*, *trnG-UCC*, *rpoC1*, *ycf3*, *trnL*-*UAA*, *trnV*-*UAC*, and *clpP*) were located in the LSC, while the *ndhA* was only present in the SSC region. In addition, the *ycf3* and *clpP* each contain two introns (**Table S3**).

### 3.2 Repeat analysis

Repetitive sequences in cp genomes play a critical role in genome evolution and rearrangements. Analysis of oligonucleotide repeat revealed that the number of repeat types varied among the nine cp genomes and presented random permutations, and most repeat sequences were within 30–39 bp (**Figure S1**). Meanwhile, the frequency of F and P repeats was greater than that of R and C repeats. The structural analysis of the repetition sequence is shown in **Figure S1**. The minimum number of repeats was found in *P. niruri* subsp. *lathyroides* (26), whereas the maximum was found in *P. niruri* (49).

SSRs, also known as microsatellites, consists of repeating units of 1–6 bp in length. 56, 98, 62, 80, 97, 54, 81, 69, and 69 SSRs were identified in *P. acidus*, *P. amarus*, *P. reticulatus*, *P. urinaria*, *P. niruri*, *P. niruri* subsp. *lathyroides*, *P. emblica*, *P. pulcher*, and *P. franchetianus*, respectively (**Table S4**). The number of SSRs showed the highest in *P. amarus* (98) and the lowest in *P. niruri* subsp. *lathyroides* (54). Most SSRs were found in LSC regions instead of SSC and IR regions (**Figure S2**). More than half of the SSRs (51.85%–66.67%) were mononucleotides with the A/T motif, followed by dinucleotides (16.25%–31.48%) with a predominant motif of AT/TA, trinucleotides (1.85%–6.25%) with a predominant motif of AAT/ATT, tetranucleotide repeats (1.61%–4.35%) with a predominant motif of AAAT/ATTT, pentanucleotides (0–1.61%), and hexanucleotides (0–1.25%) that only exist in the cp genome of *P. urinaria*.

### 3.3 Codon usage bias of cp genomes

**Figure 2:**
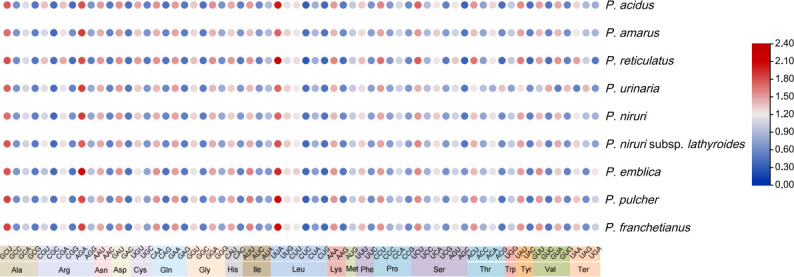
The RSCU values of nine *Phyllanthus* cp genomes. Color key: the red values indicate higher RSCU values, and the blue values indicate lower RSCU values (For interpretation of the references to color in this Figure legend, the reader is referred to the web version of this article).

The analyses of RSCU provide information about the encoding frequency of codons for an amino acid. There were 64 codons in the coding sequence of nine *Phyllanthus* species genes, among which 61 codons encoded 20 amino acids, and the other three codons (UAA, UAG, and UGA) were stop codons (**Table S5**). Amino acid frequency analyses revealed that the highest frequencies were leucine and isoleucine, whereas cysteine was a rare amino acid. The codon exhibited a strong bias toward an A or T at the third position. An RSCU value below 1.00 indicates that the codon usage frequency is lower than expected, whereas an RSCU value above 1.00 indicates that the codon usage frequency is higher than expected. In this study, the RSCU values of 30 codons were greater than 1, whereas the RSCU value of 32 codons was less than 1, and 2 codons were equal to 1 (**Figure 2**).

Moreover, the results showed that the GC content of synonymous third codon positions (GC3s) is closely related to codon bias, and the values of GC3s ranged from 25.0% to 31.1%, suggesting that the genus *Phyllanthus* had a greater preference for the A/U ending codons. And the GC content of these cp genomes was highly conserved. In addition, the values for the effective number of codons ranged from 48.43% to 52.93%. Both the codon adaptation index and optimal frequency were less than 0.5. These findings indicated a slight bias toward codon usage in the nine *Phyllanthus* species.

### 3.4 Inverted repeats

**Figure 3:**
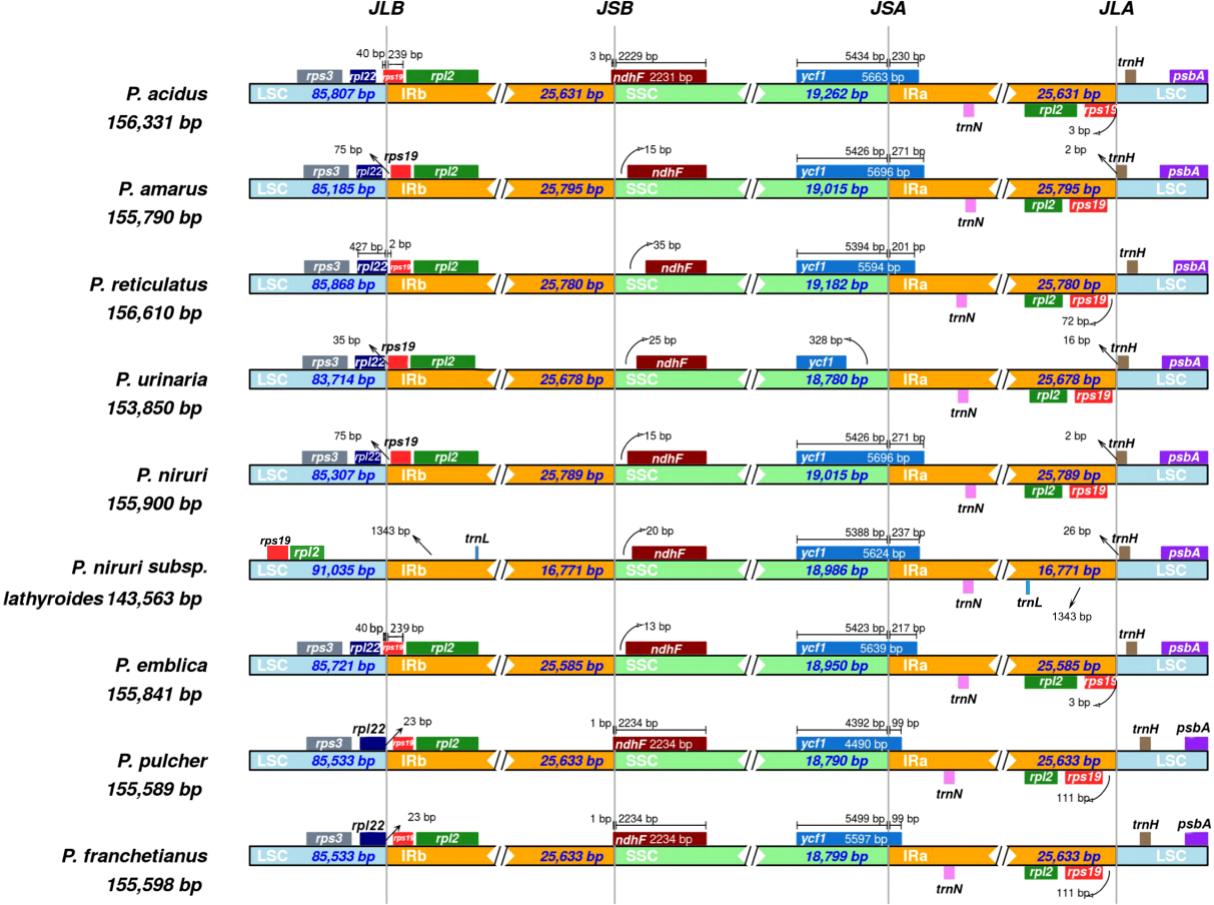
Comparisons of the borders of LSC, SSC, and IRa/b regions among the nine *Phyllanthus* cp genomes. The numbers represent the distance between the gene ends and the border sites, and the numbers below represent the length of the LSC, SSC, and IRa/b regions.

Expansion and contraction at the borders of IR regions are common evolutionary phenomena that may explain variations in the size of cp genomes. As illustrated in **Figure 3**, the *rps3* genes existed entirely in the LSC regions of all species, and *rpl2* existed entirely in the IR region except for *P. niruri* subsp. *lathyroides.* A truncated copy of the *rpl22* gene was observed at the junction of LSC/IRb in three species (*P. reticulatus*, *P. pulcher*, and *P. franchetianus*), which starts in LSC regions and integrates into the IRb region with a size ranging from 2 to 23 bp, whereas the remaining six species were present entire in the LSC region. Another truncated copy of the *rps19* gene was found at the junction of IRa/SSC in two species (*P. acidus* and *P. emblica*). Notably, *rps19* exists entirely in the LSC region for *P. niruri* subsp*. lathyroides*, and *rps19* is present entirely in the IR region in the remaining six species. Besides, the *ndhF* gene was found in the SSC regions except for three species (*P. acidus*, *P. pulcher*, and *P. franchetianus*), which start in the SSC regions and integrate into the IRb region in those species. Moreover, the *ycf1* gene was observed at the IRa/SSC junction except for *P*. *urinaria*. In all other species, the *ycf1* gene starts in SSC regions and integrates into the IRa. However, in *P*. *urinaria*, the *ycf1* gene is completely present in the SSC region. Both *psbA* and *trnN* exist entirely in the LSC and IRa, respectively. Notably, the *trnL* gene exists only in the IR region of *P. niruri* subsp. *lathyroides*. These results show that the cp genomes of nine *Phyllanthus* species display a unique IR contraction and expansion type.

### 3.5 Genome comparison and nucleotide diversity

**Figure 4:**
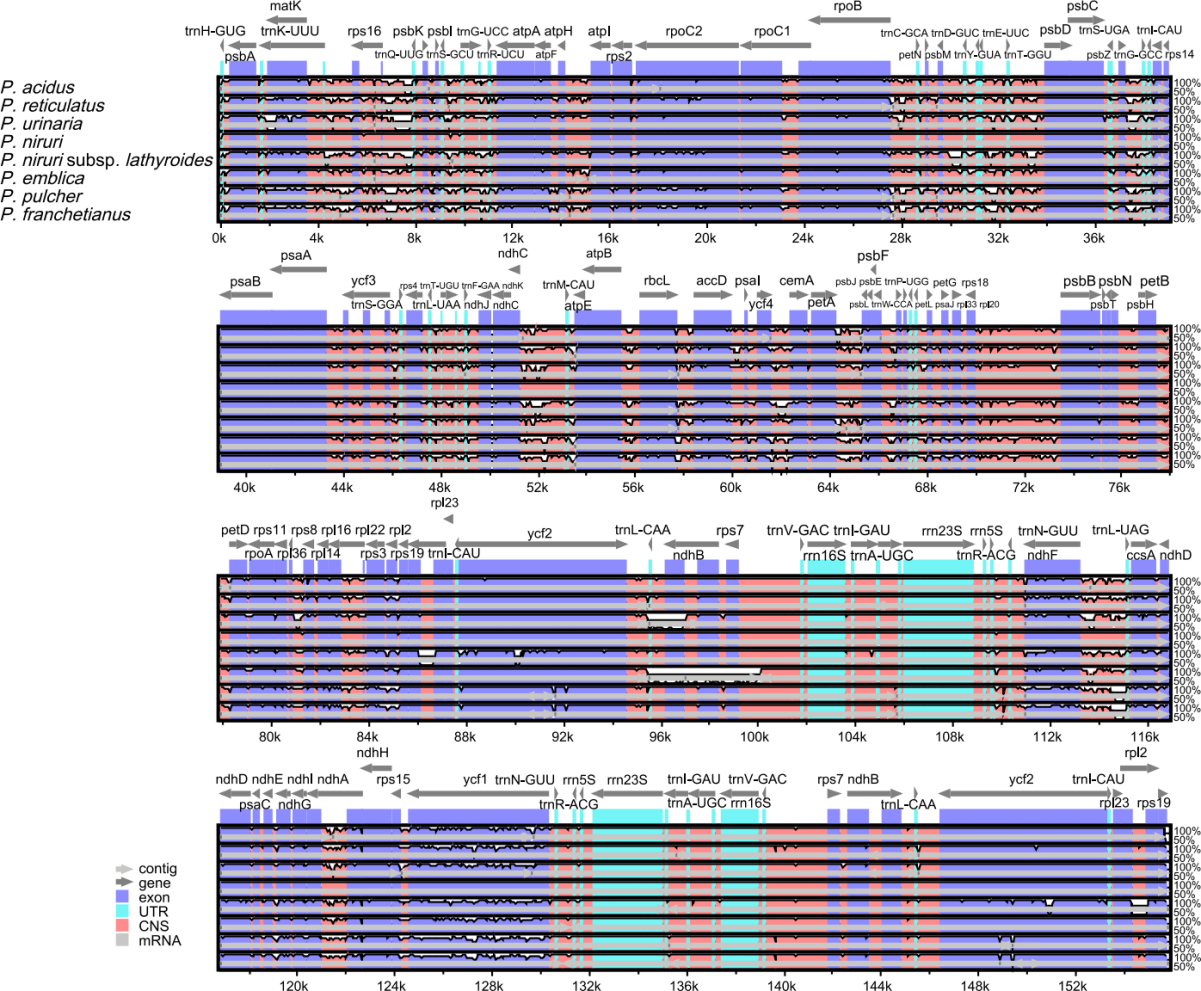
Comparison of nine cp genomes using *P*. *amarus* annotation as a reference. The vertical scale indicates the percentage of identity, ranging from 50% to 100%. The horizontal axis shows the coordinates within the cp genome. Genome regions are color-coded as exons, introns, and IGS, and the gray arrows indicate the direction of transcription of each gene. Annotated genes are displayed along the top.

A comparison of overall sequence variation showed that the cp genome of *Phyllanthus* is quite different. The sequence divergence of IR regions was lower than that of SSC and LSC regions, and the coding region was more conserved than the non-coding regions. Except for the more remarkable mutations in the *ndhF*, *ycf1*, and *ycf2* genes, all protein-coding genes showed a highly conserved character. The highest divergence was mainly found in intergenic spacers (IGS), such as *rps16-trnQ-UUG*, *trnS-GCU-trnG-UCC*, *trnG-UCC-trnR-UCU*, *trnE-UUC-trnT-GGU*, *trnD-GUC-trnY-GUA*, *trnT-UGU-trnL-UAA*, *trnL-CAA-ndhB*, *trnN-GUU-trnR-ACG*, and *rps15-ycf1* (**Figure 4**). Besides, the sliding window analysis demonstrated that the nine regions, including *rps16*, *trnS-GCU-trnG-UCC*, *trnG-UCC-trnR-UCU*, *petA-psbJ*, *rps3*, *rrn5S-trnR-ACG*, *ndhF*, *ndhE-ndhG*, and *ycf1*, had higher nucleotide diversity values (Pi > 0.05) (**Figure S3**). The results above show that 12 highly variable sites (*rps16-trnQ-UUG*, *trnS-GCU-trnG-UCC*, *trnG-UCC-trnR-UCU*, *trnE-UUC-trnT-GGU*, *trnD-GUC-trnY-GUA*, *trnT-UGU-trnL-UAA*, *trnL-CAA-ndhB*, *trnN-GUU-trnR-ACG*, *rps15-ycf1*, *petA-psbJ*, *rrn5S-trnR-ACG*, and *ndhE-ndhG*) might be able to be used as molecular markers to identify *P. urinaria* and its contaminants.

### 3.6 Species authentication analysis based on IGS

IGS regions are the most commonly used markers for phylogenetic studies at plant taxonomic levels, as they are regarded as more variable and may provide more phylogenetically informative characters. To find candidate sequences for identifying *P*. *urinaria* and its adulterants, 12 IGS were extracted from 13 *Phyllanthus* species using PhyloSuite v1.2.2. And each of them is subject to ML analyses in IQtree. As shown in **Figure S4.1**-**4.12**, *P. urinaria* could be distinguished from its common adulterants based on *trnS-GCU-trnG-UCC*, *trnT-UGU-trnL-UAA*, and *petA-psbJ*, whereas the remaining IGS cannot be distinguished, and the bootstrap values for the relationship among these clades were weak (<70%). Furthermore, the ML phylogenetic tree was also inferred using a combination of these three IGS. The results (**Figure S5**) showed that *P. urinaria* (Genbank OP009346) was located in independent branches and that there was a well-supported sister relationship between *P. urinaria* (Genbank OP009346) and *P. amarus* (Genbank OP009344) + *P. urinaria* (Genbank NC060522) + *P. niruri* (Genbank OP009347). These results indicated that combining three IGS could effectively discriminate *P. urinaria* from its common adulterants.

### 3.7 Phylogenetic analysis and divergence time analysis

**Figure 5:**
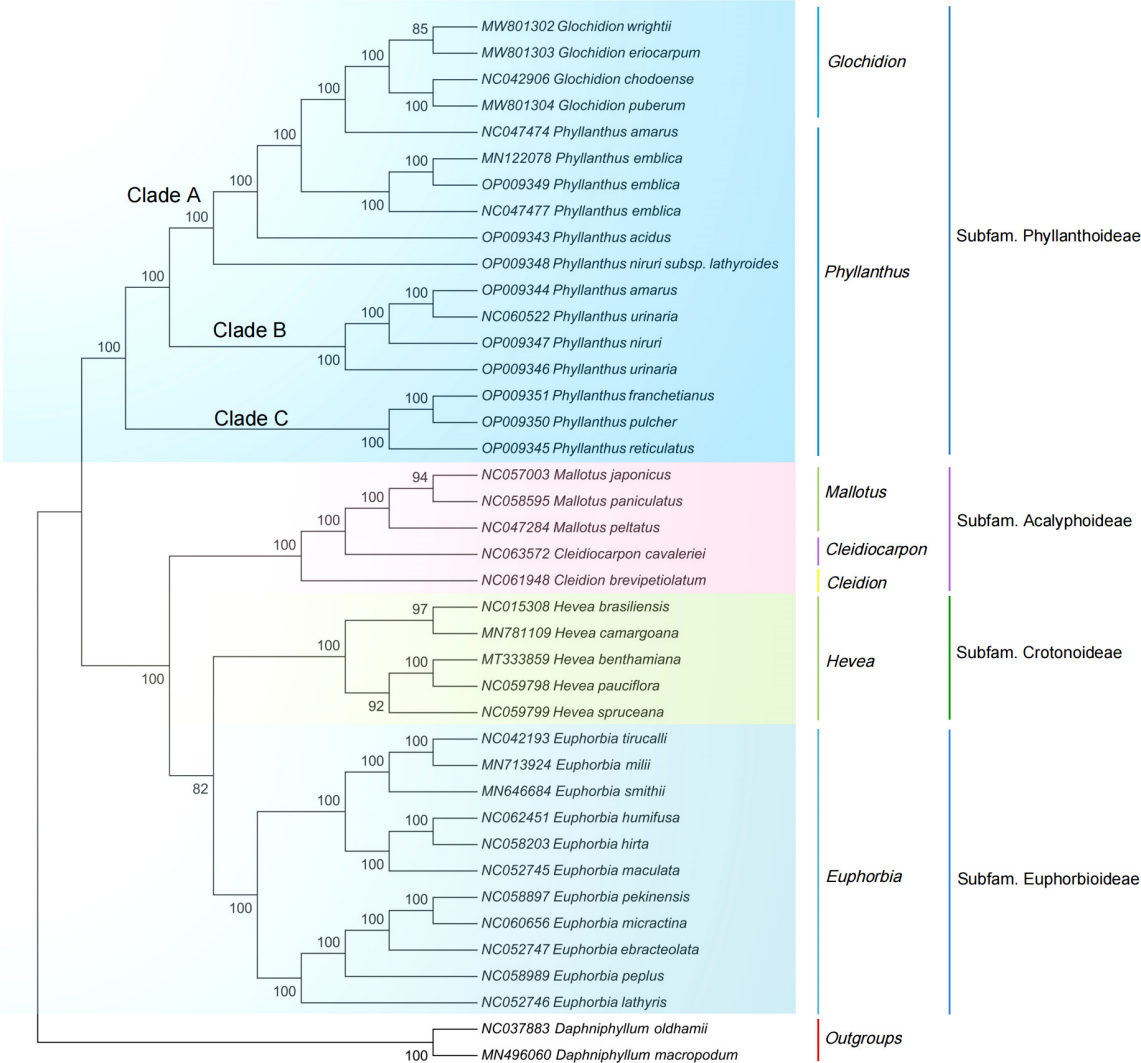
Maximum likelihood phylogenetic tree based on complete cp genomes. *Daphniphyllum oldhamii* and *D. macropodum* were used as outgroups. Numbers at nodes are bootstrap support values.

The ML and NJ phylogenetic trees were inferred using 40 species, with two *Daphniphyllum* species as outgroups. The consensus trees obtained from the inference analyses showed that most nodes resolved with high support (**Figure 5**, **Figure S6**). The phylogenetic trees generated by the ML and NJ alignments have similar topologies. Each subfamily of the Euphorbiaceae family forms a monophyletic clade. Acalyphoideae and Euphorbioideae + Crotonoideae were sister taxa within the four subfamilies, and Phyllanthoideae was a sister group to the clade containing Acalyphoideae + Crotonoideae + Euphorbioideae. In addition, the subfamily Phyllanthoideae is further divided into three clades: (i) clade A, including *Glochidion wrightii* (Genbank MW801302), *G. eriocarpum* (Genbank MW801303), *G. chodoense* (Genbank NC042906), *G. puberum* (Genbank MW801304), *P. amarus* (Genbank NC047474), *P. emblica* (Genbank MN122078, Genbank OP009349, Genbank NC047477), *P. acidus* (Genbank OP009343), and *P. niruri* subsp*. lathyroides* (Genbank OP009348); (ii) clade B, including *P. amarus* (Genbank OP009344), *P. urinaria* (Genbank NC060522), *P. niruri* (Genbank OP009347), and *P. urinaria* (Genbank OP009346); (iii) clade C, including *P. franchetianus* (Genbank OP009351), *P. pulcher* (Genbank OP009350), and *P. reticulatus* (Genbank OP009345).

**Figure 6:**
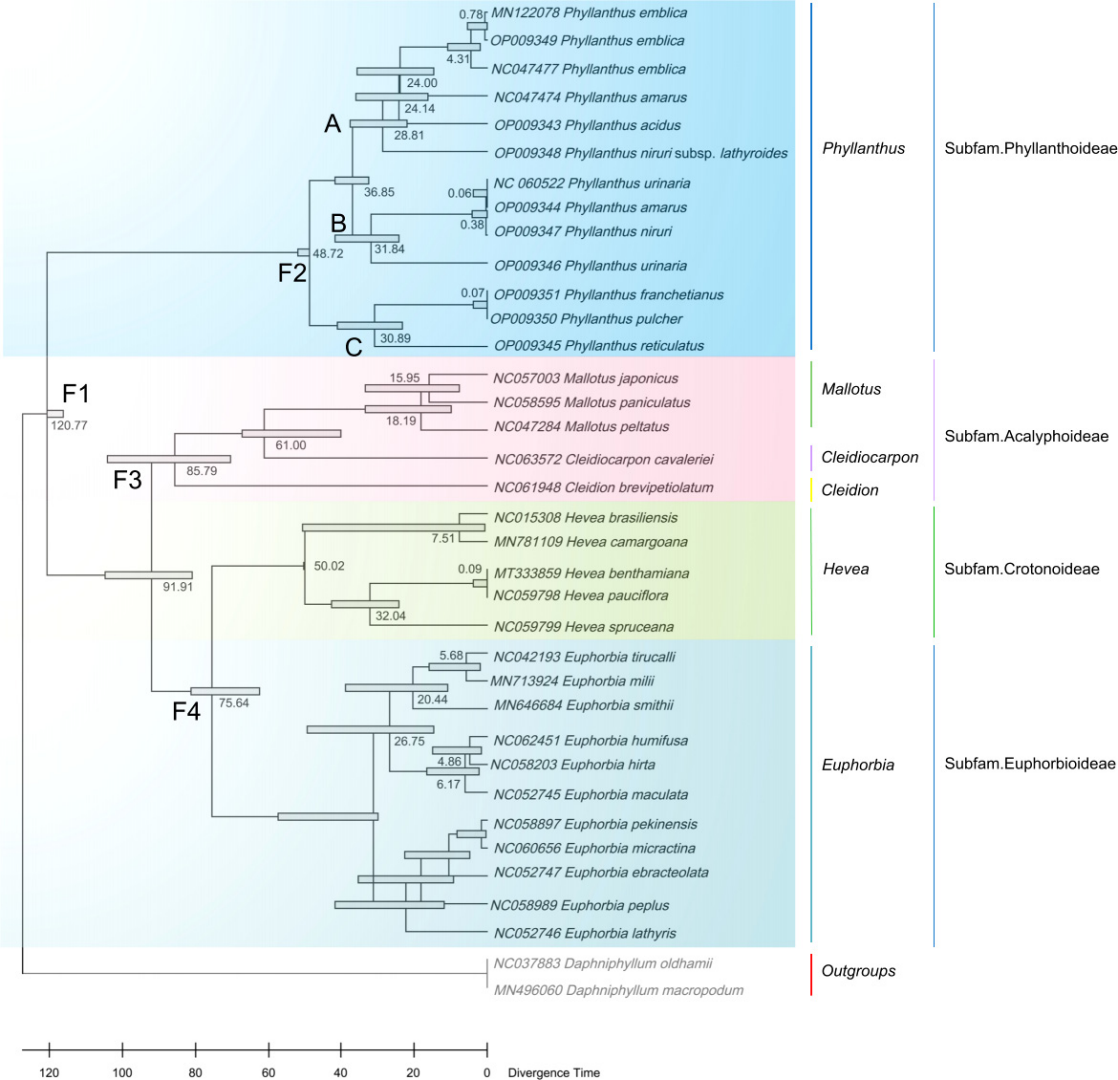
Divergence times estimation based on cp genomes. The node ages are given for each node.

In addition, 34 cp genomes of Euphorbiaceae family plants (including 13 *Phyllanthus* species) and two outgroups are used to estimate the divergence times. *Phyllanthus* were estimated to have originated 48.72 million years ago (Ma). The two main lineages, clade A + clade B and clade C, seem to have radiated since the Oligocene (clade A + clade B: 36.85 Ma; clade C: 30.89 Ma; **Figure 6**). The extant subfamilies of the Euphorbioideae and Acalyphoideae shared a common ancestor at the beginning of the Cretaceous (91.91 Ma), while the split between the Euphorbioideae and Crotonoideae is estimated to have occurred at 75.64 Ma. Moreover, the divergence times of the *Daphniphyllum* occurred at 120.77 Ma, having shared a common ancestor with the Euphorbiaceae.

## 4 Discussion

### 4.1 Cp genome structure and comparative analysis

In the present study, the cp genomes of nine species of *Phyllanthus* were assembled *de novo* and analyzed comparatively. The observed genome size is within the size range known for most angiosperms, ranging from 107 kb in *Cathaya argyrophylla* (Pinaceae) to 218 kb in *Pelargonium* (geraniums; Geraniaceae) (Daniell et al., 2016). Besides, our findings revealed that the total length, GC content, and gene composition of the cp genomes were almost identical in all species. Previous studies have shown that the cp genome of angiosperms is highly conserved at the genus level (Khan et al., 2020; Villanueva-Corrales et al., 2021; Feng et al., 2022). Moreover, previous research demonstrated that the *rps19* gene existed in the IR region (Ahmed et al., 2012; Wang et al., 2022). In contrast, the *rps19* of *P. niruri* subsp*. lathyroides* is located entirely in the LSC Region, possibly due to IR contraction. The same pattern was reported in *Anchomanes hookeri*, and *Peucedanum* (Henriquez et al., 2020; Liu et al., 2022).

### 4.2 Species identification and phylogenetic analysis

Previous molecular studies of the *Rheum*, *Hedyotis*, and *Curcuma* species showed that cp genetic markers had high identification capabilities (Zhou et al., 2018; Gui et al., 2020; Yik et al., 2021). Our study revealed that three regions (*trnS-GCU-trnG-UCC*, *trnT-UGU-trnL-UAA*, and *petA-psbJ*) might be potential molecular markers for identifying *P. urinaria* and its common adulterants. Bouman et al. (2021) found that the *trnS-GCU-trnG-UCC* could distinguish *Phyllanthus* species. Notably, Zhang et al. (2021) also found that the *trnS-GCU-trnG-UCC* could be potential molecular markers for distinguishing *Alpinia* species. Moreover, *trnT-UGU-trnL-UAA* or *petA-psbJ* were reported as potential markers for other species identification (Dong et al., 2021; Wu et al., 2021). Although these previous studies revealed that universal DNA barcode (e.g., *psbA-trnH*) could differentiate *P. urinaria* from their related species (Srirama et al., 2010; Inglis et al., 2018), some common adulterants were not included in these studies. Furthermore, the comparative analysis showed that the screened IGS exhibited higher variability than *psbA-trnH*. Theoretically, these IGS could differentiate nine selected species, whereas a much more thorough investigation of identification accuracy and amplification efficiency is required, as well as more experimental evidence.

Moreover, ML analysis demonstrated that *P. urinaria* (Genbank OP009346) was located in independent branches in the phylogeny and strongly supported the sister relationship between *P. urinaria* (Genbank OP009346) and the well-supported clade (*P. amarus*, Genbank OP009344; and *P. niruri*, Genbank OP009347). The results indicated that the cp genome could discriminate *P. urinaria* from *P. amarus* and *P. niruri*, which was supported by the findings of other researchers based on ITS, *matK*, *psbA-trnH*, *trnL*, and *trnL-trnF* (Inglis et al., 2018). However, the samples of *P. urinaria* (Genbank OP009346) and *P. urinaria* (Genbank NC060522) were not recovered as monophyletic and were placed in different branches. In the previous study, some researchers found that intraspecific diversity existed in *Isodon rubescens* and *Artemisia argyi* from different geographical areas (Zhou et al., 2022; Chen et al., 2022). Therefore, the difference in geographical origins may explain why the two species are split in these clades. Besides, both NJ and ML analyses found strong support for a sister relationship between *P. reticulatus* (Genbank OP009345) and *P. pulcher* (Genbank OP009350), which agreed with the findings of Hidalgo et al. (2020) based on *trnK-matK*, *matK*, ITS, and *matK+*ITS (Pagare et al., 2016). In a previous study, Rehman et al. (2021) also found that the polymorphic protein-coding genes, including *rpl22*, *ycf1*, *matK*, *ndhF*, and *rps15*, may help reconstruct the high-resolution phylogenetic tree of the family Phyllanthaceae. In general, our results provide a valuable reference and a foundation for using cp genomes in species identification and aid in the understanding of the phylogeny of *Phyllanthus*.

### 4.3 Divergence time of *Phyllanthus*

According to divergence time estimates, the early divergence of *Phyllanthus* occurred at approximately 48.72 Ma during the early Eocene, which is congruent with other studies (Kawakita and Kato, 2009; Welzen et al., 2015). Since the late Eocene, the previous study reported that the global climate started to have a notable change; as the humidity and precipitation gradually increased (Zachos et al., 2001) and slowly cooled within this timeframe. These climate changes may have promoted the dispersals/migrations, and diversification of land plants (Zuo et al., 2017). In addition, Dynesius and Jansson (2000) and Zachos et al. (2001) also found that the temperature increase affected various plant and animal communities and groups at the Oligocene/Miocene boundary (~23 Ma). Among the effects of climate change, there was likely increased speciation in the niches that opened after the end of the climatic fluctuations (Bouman et al., 2021). Therefore, we can conclude that the climatic changes may have contributed to the diversification of *Phyllanthus* during the Eocene.

## 5 Conclusion

In the present study, the complete cp genomes of nine species of *Phyllanthus* were *de novo* assembled from high throughput sequencing reads, and the cp genomes of *P. acidus*, *P. reticulatus*, *P. niruri*, *P. niruri* subsp. *lathyroides*, *P. pulcher*, and *P. franchetianus* were reported for the first time. These cp genomes were generally conserved and exhibited similar gene content and genomic structure. Three highly variable cp loci, including *trnS-GCU-trnG-UCC*, *trnT-UGU-trnL-UAA*, and *petA-psbJ* were identified and could serve as candidate markers for identifying *P. urinaria* and its common adulterants. Meanwhile, the complete cp genome was considered a reliable molecular marker for identifying these species, which may have virtual significance for protecting their diversity and making management decisions for this species. The divergence of *Phyllanthus* from ancestral taxa occurred in the early Eocene, which might be due to geological and climatic changes. In conclusion, our study provides a powerful tool and valuable scientific reference for the safety and effectiveness of clinical drug use, and it also contributes to the bioprospecting and conservation of *Phyllanthus* species.

## Data availability statement

The data presented in the study are deposited in the GenBank repository, accession numbers were from OP009343 to OP009351.

## Author contributions

HF, YL, and PZ participated in the conception and design of the research. HF, GD and BL collected the species. HF and GD are responsible for analyzing and processing data. HF wrote the manuscript. The paper was revised by YL and PZ. All authors contributed to the article and approved the submitted version.
