# Supplementary material for: Application of chloroplast genome in the identification of Phyllanthus urinaria and its common adulterants
Source: Front Plant Sci. 2023 Jan 6;13:1099856. doi: 10.3389/fpls.2022.1099856 (PMC9853280; doi:10.3389/fpls.2022.1099856)
Supplement: Supplementary file 1 [file DataSheet_1.pdf]

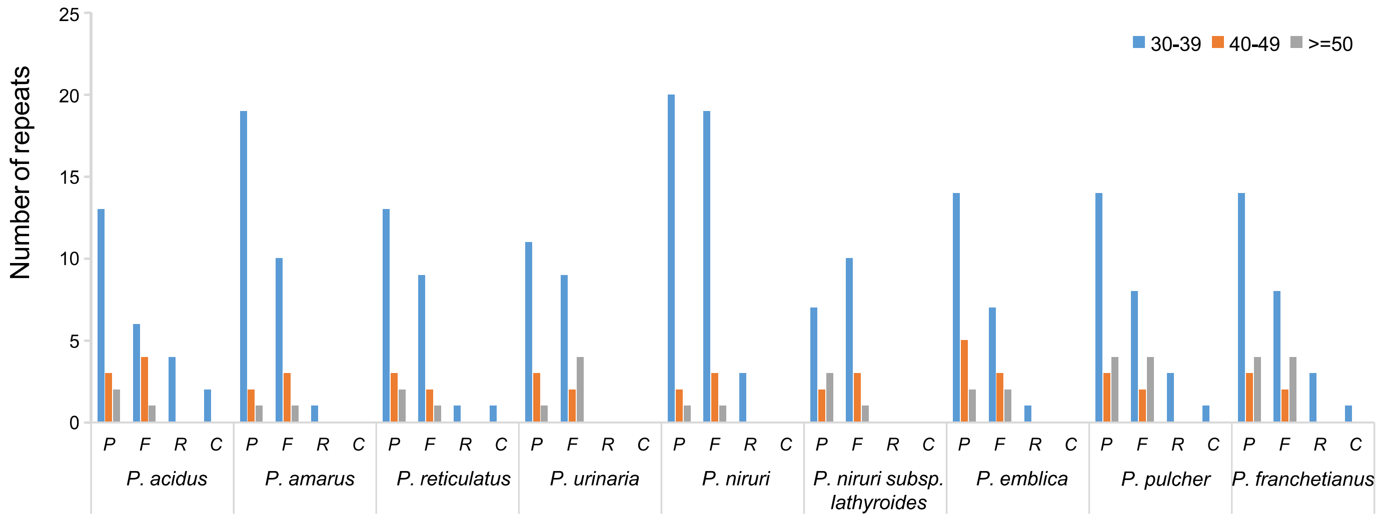

Fig. S1. Repeat sequences detected in the cp genome of Phyllanthus. P, F, R, and C indicate the repeat types: P (Palindromic repeats), F (Forward repeats), R (Reverse repeats), and C (Complement repeats).

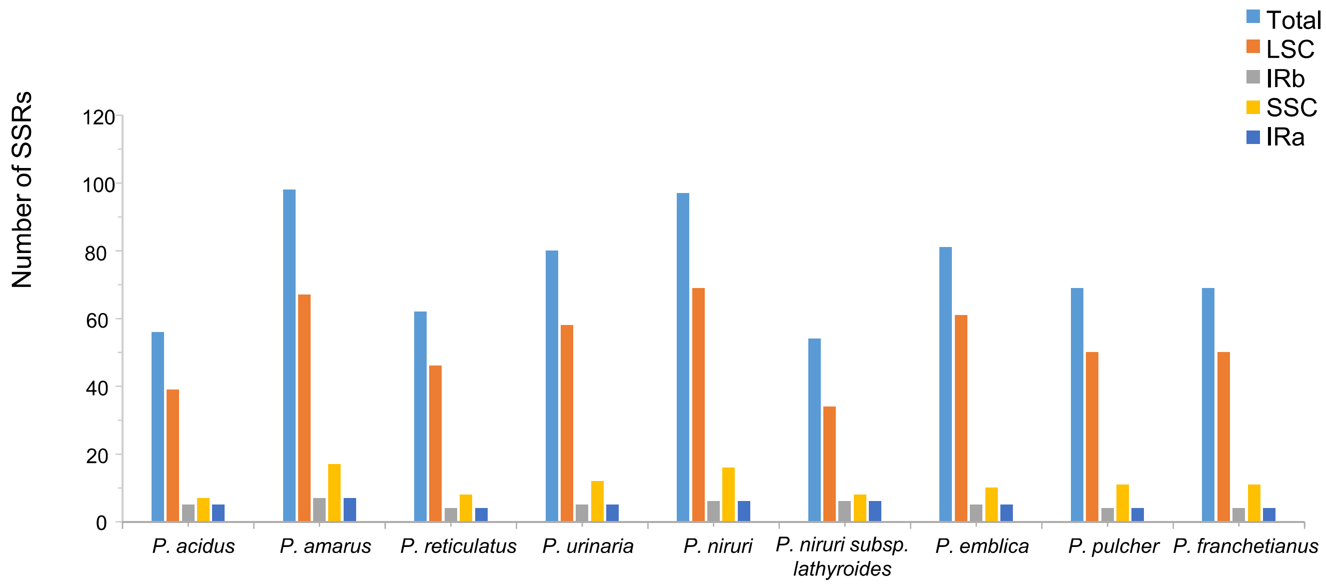

Fig. S2. Distribution of SSRs in LSC, IRa/IRb, and SSC.

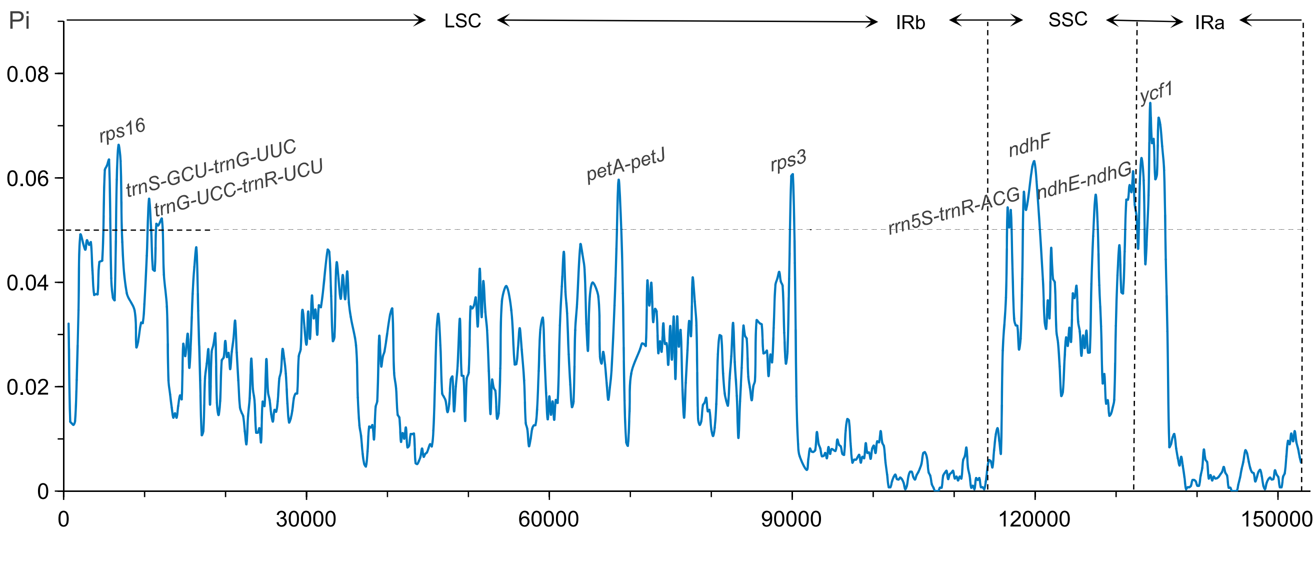

Fig. S3. Sliding window analysis of Phyllanthus cp genomes. The X-axis represents the midpoint of the window; the Y-axis represents nucleotide diversity values.

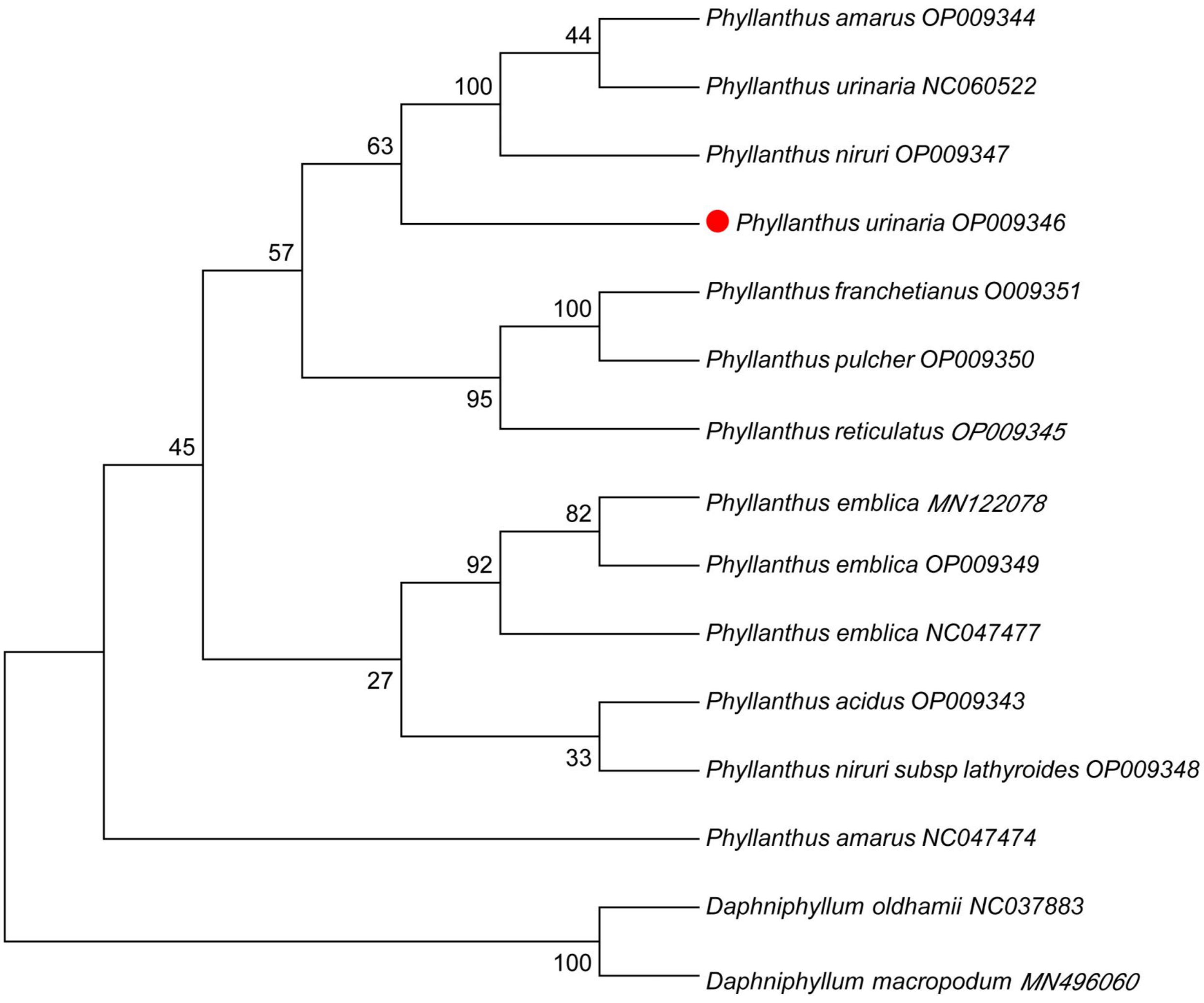

Fig. S4.1. Phylogenetic tree constructed using ML method based on *rps16-trnQ-UUG.*

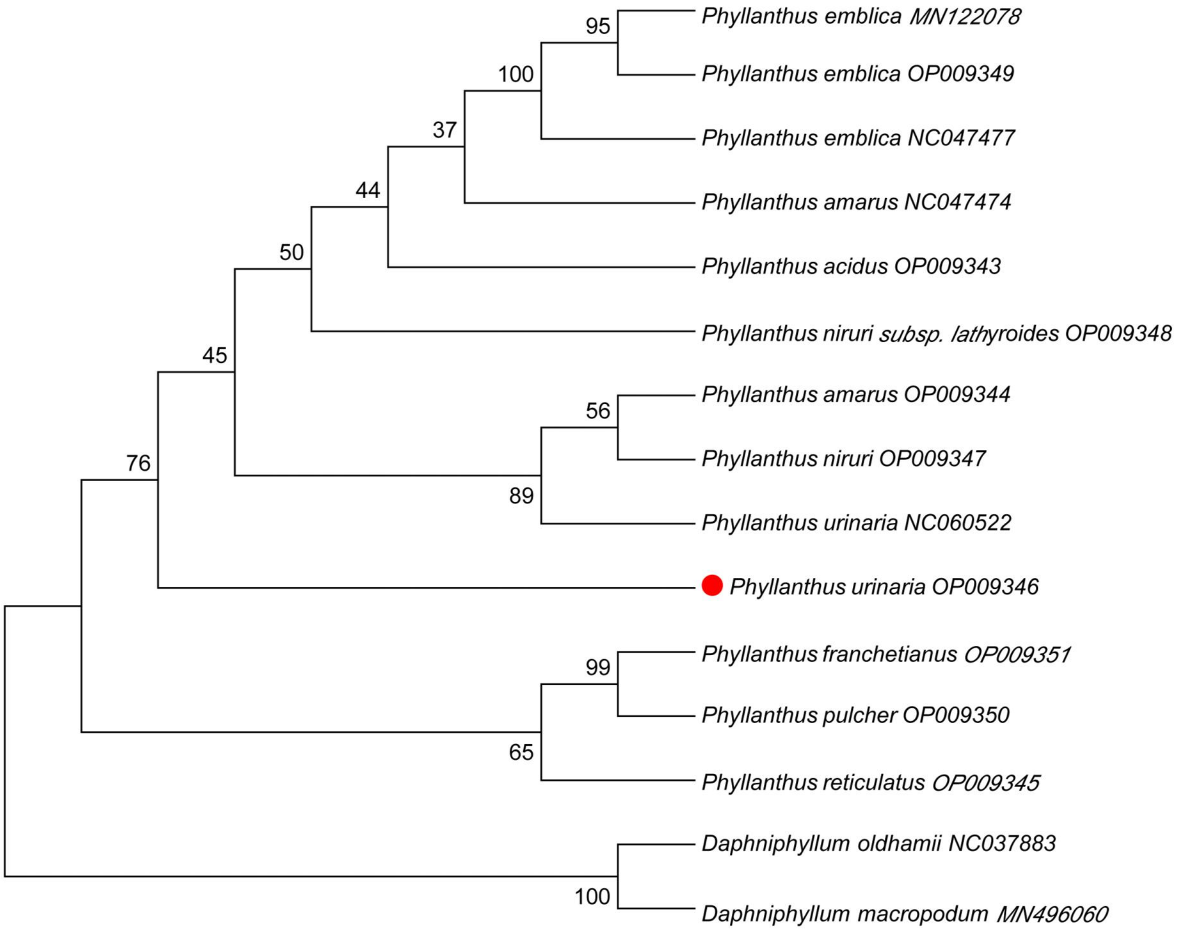

Fig. S4.2. Phylogenetic tree constructed using ML method based on *trnS-GCU-trnG-UCC.*

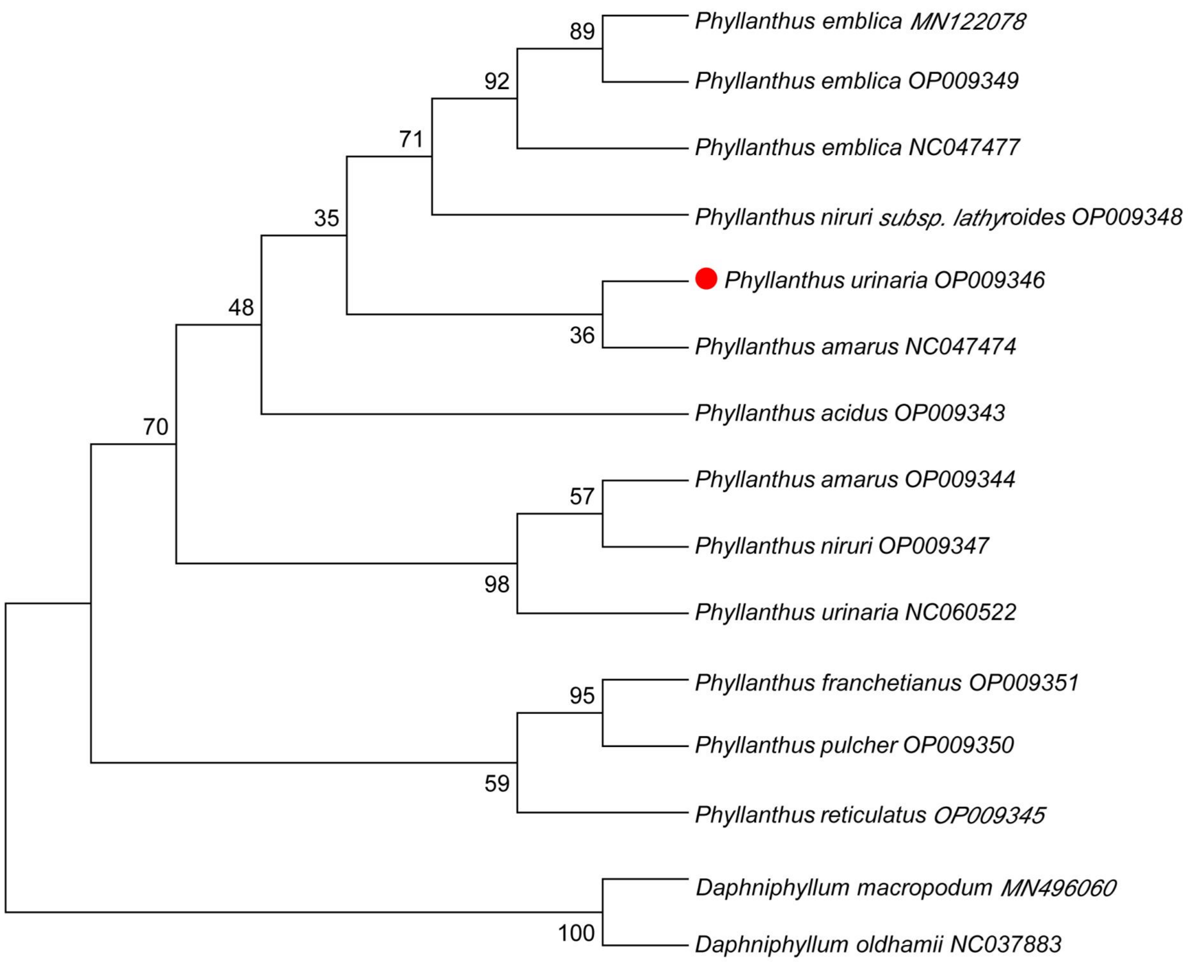

Fig. S4.3. Phylogenetic tree constructed using ML method based on *trnG-UCC-trnR-UCU.*

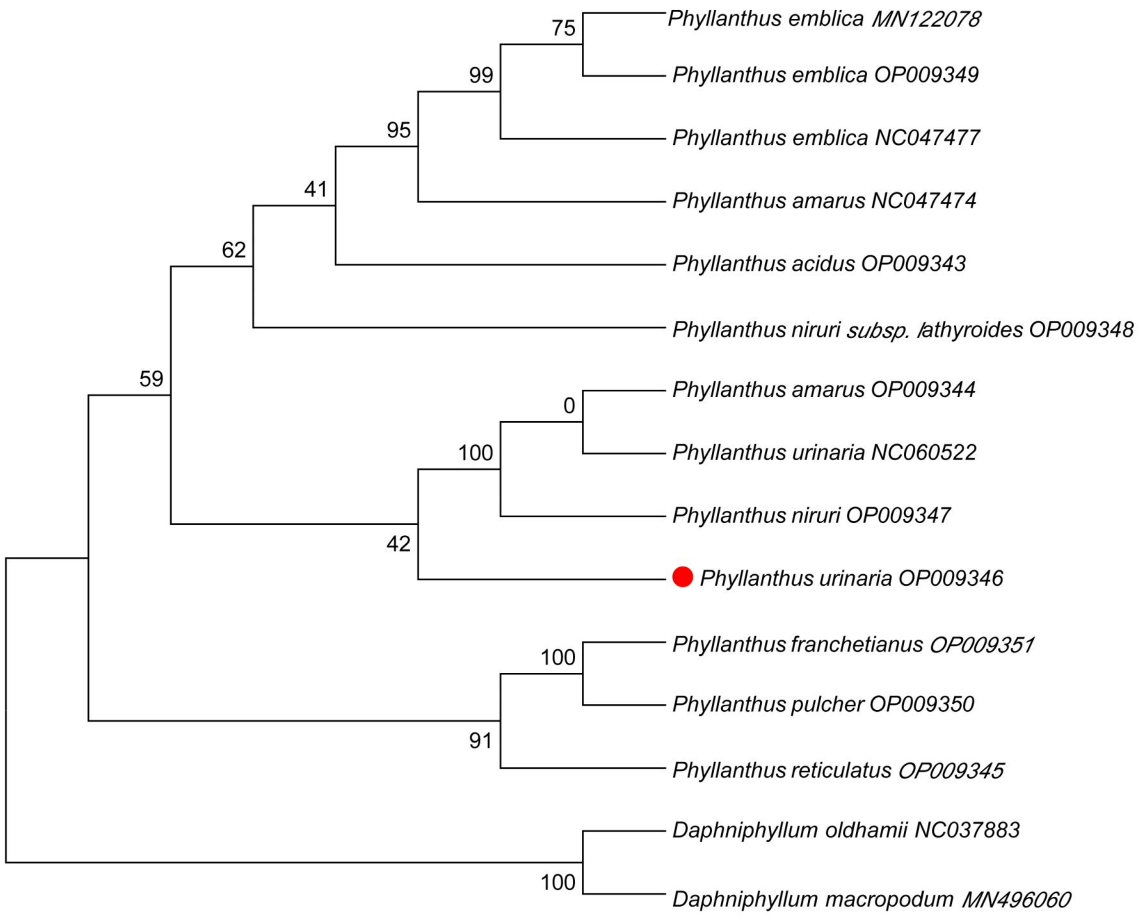

Fig. S4.4. Phylogenetic tree constructed using ML method based on *trnE-UUC-trnT-GGU.*

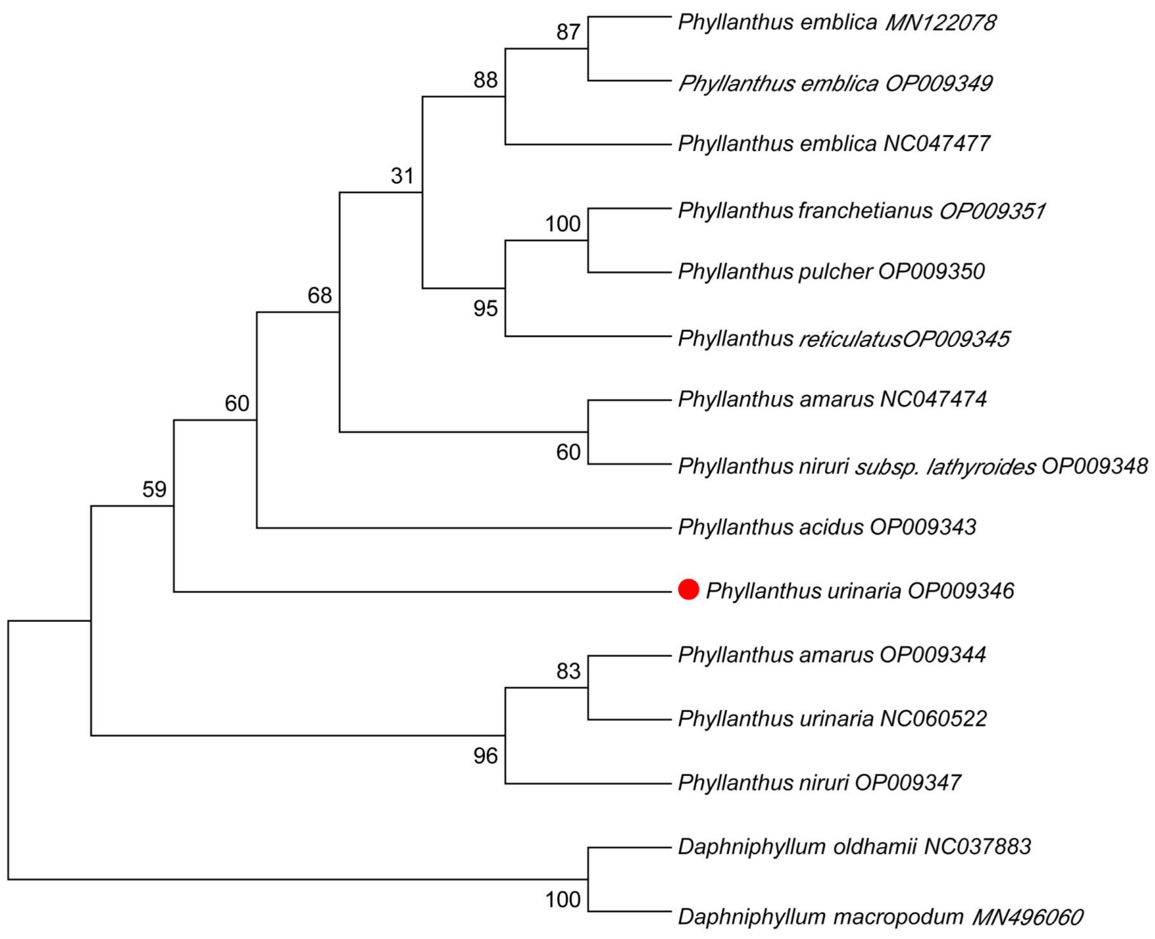

Fig. S4.5. Phylogenetic tree constructed using ML method based on *trnD-GUC-trnY-GUA*.

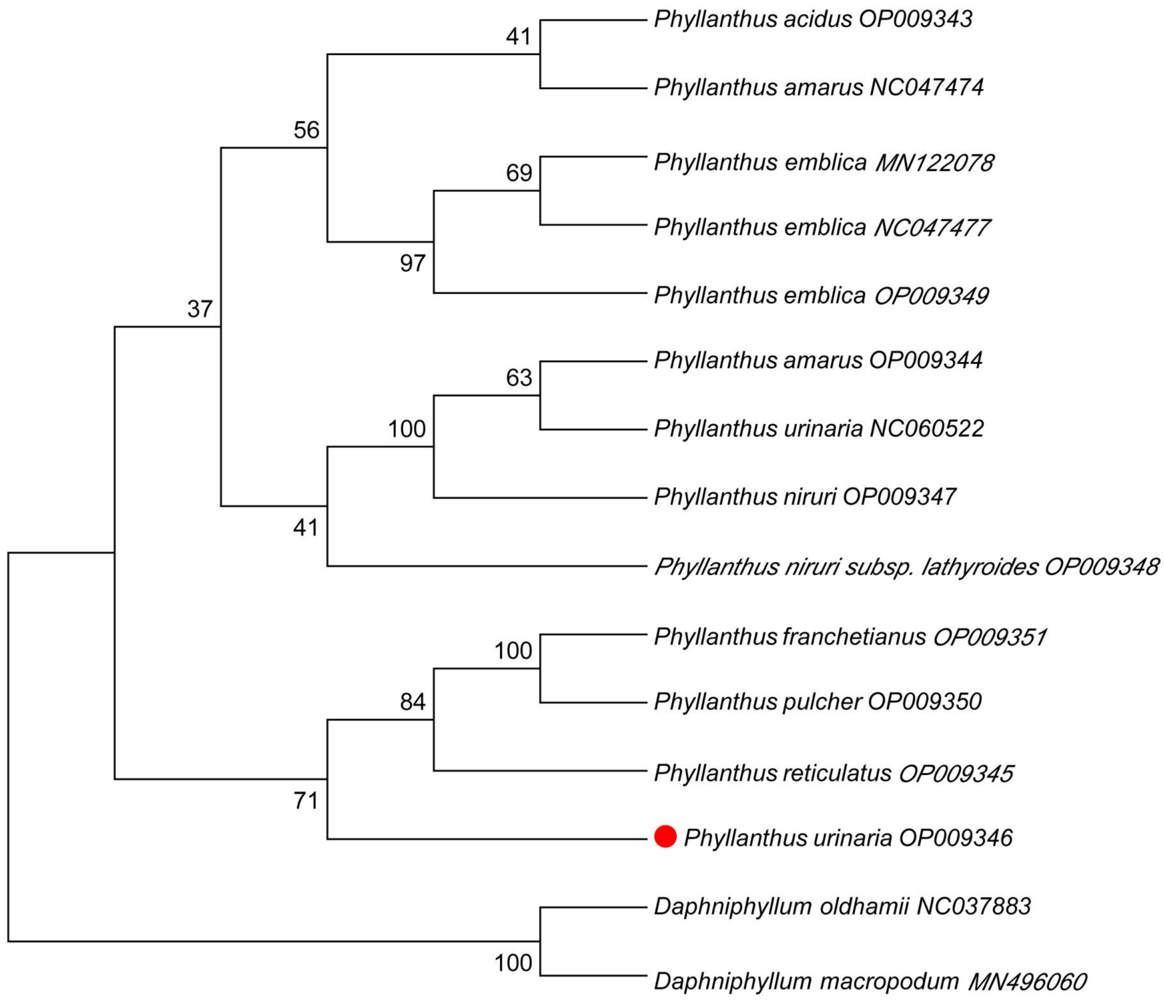

Fig. S4.6. Phylogenetic tree constructed using ML method based on *trnT-UGU-trnL-UAA*.

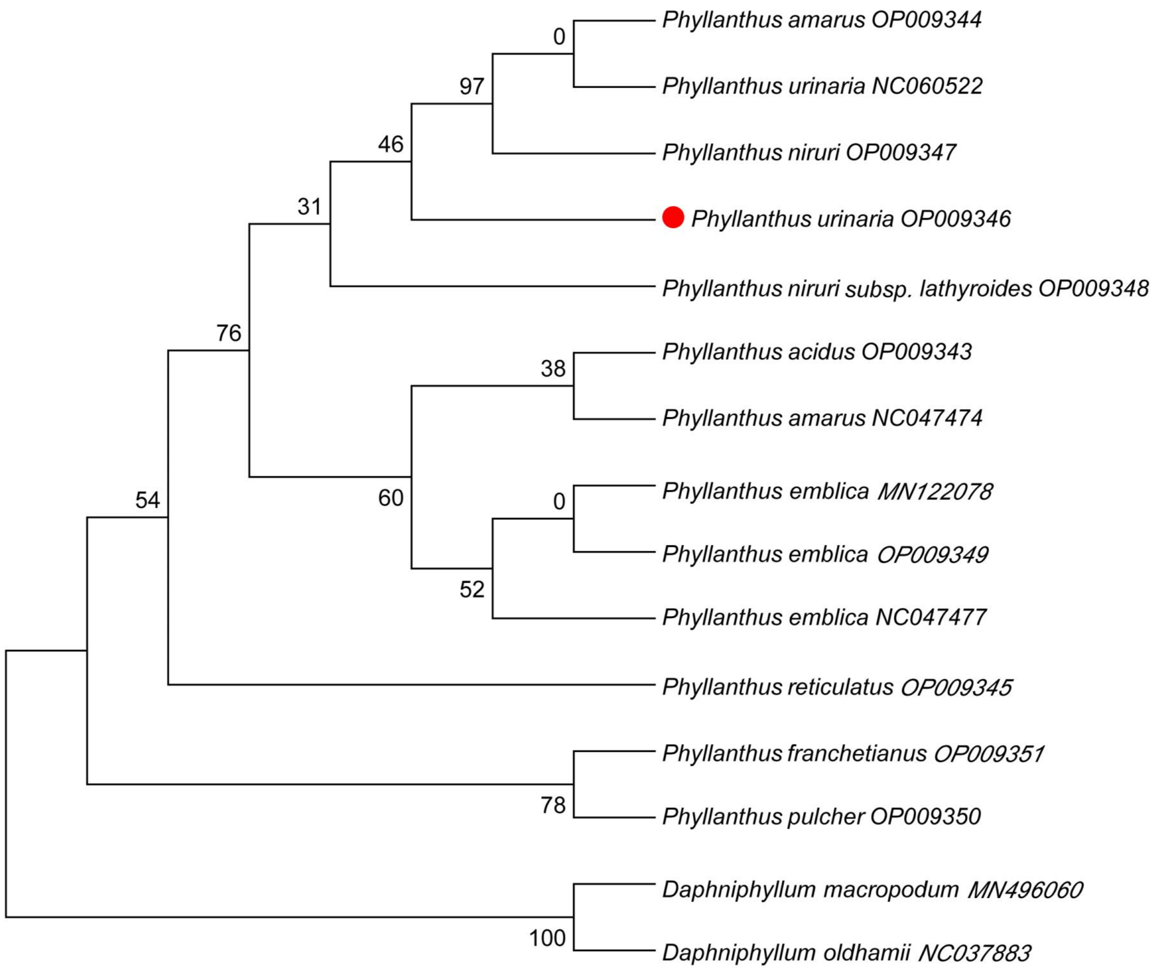

Fig. S4.7. Phylogenetic tree constructed using ML method based on *trnL-CAA-ndhB.*

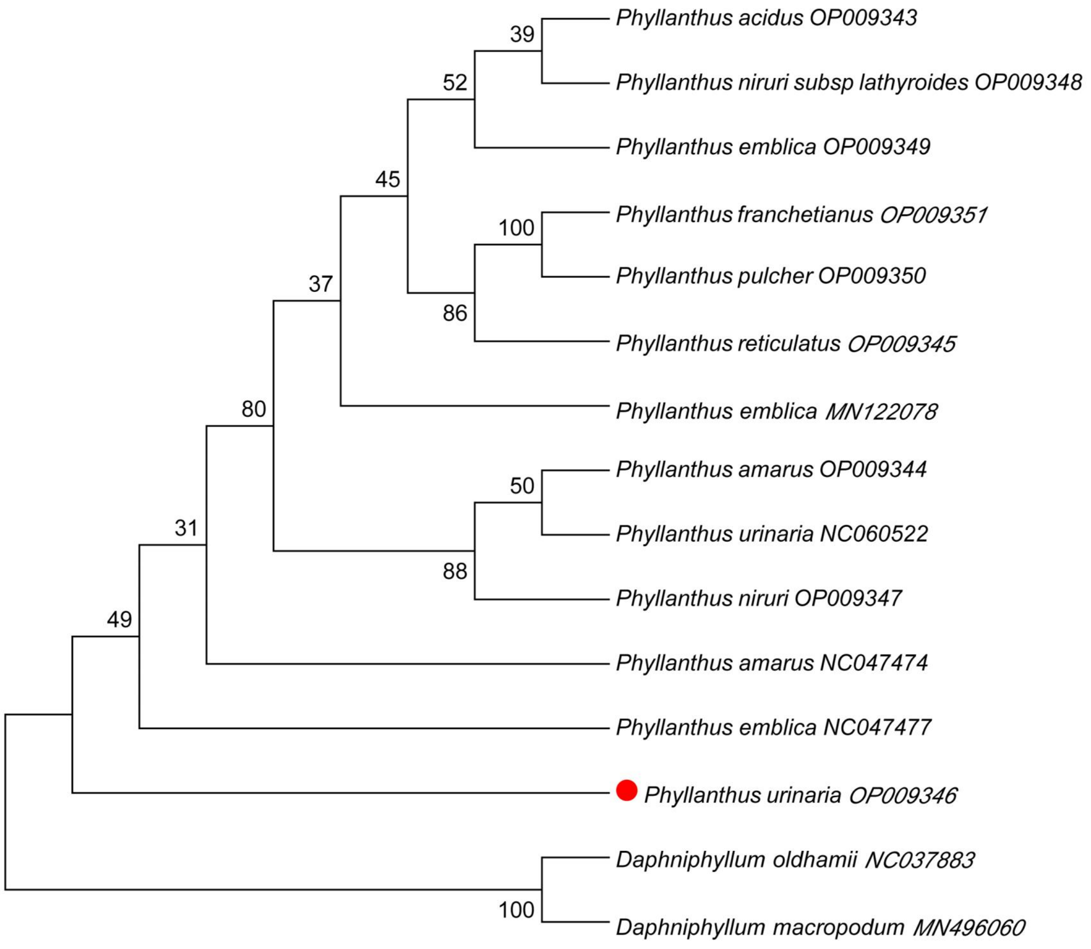

Fig. S4.8. Phylogenetic tree constructed using ML method based on *trnN-GUU-trnR-ACG.*

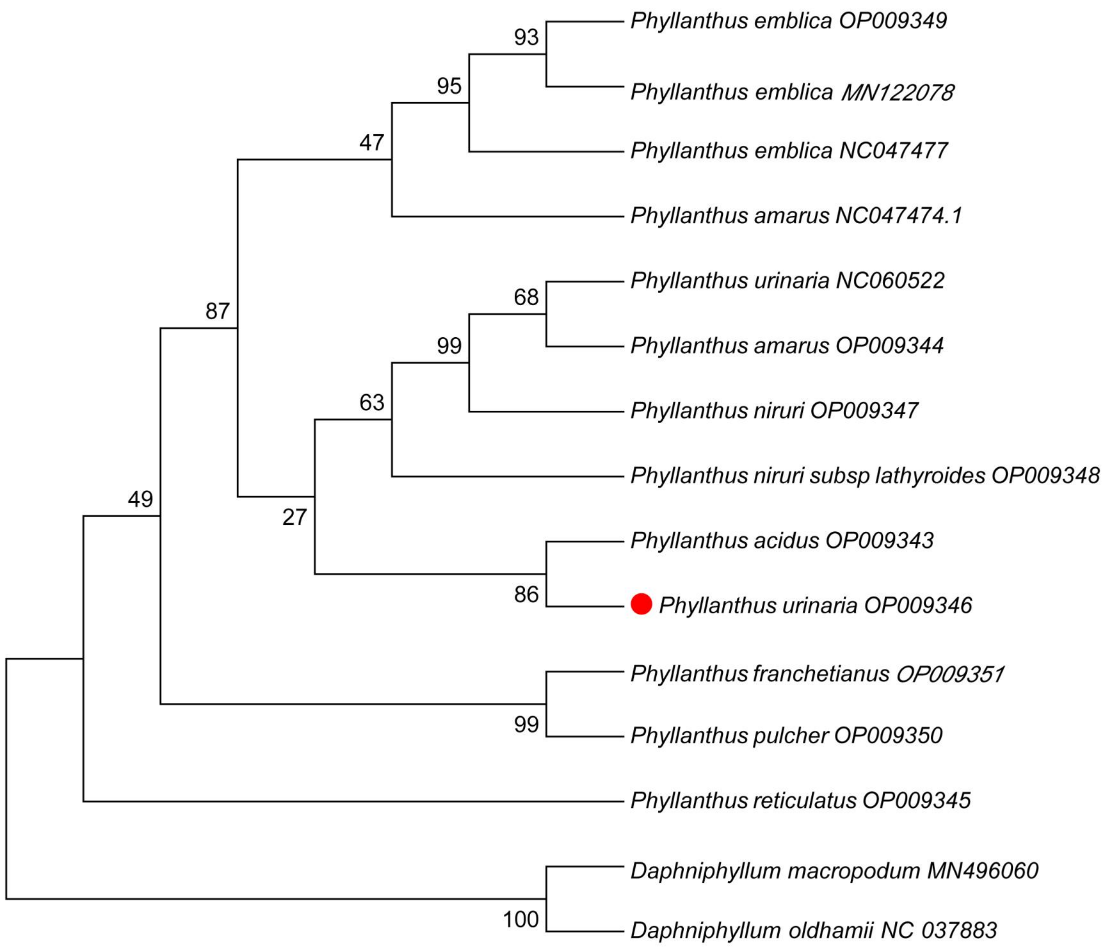

Fig. S4.9. Phylogenetic tree constructed using ML method based on *rps15-ycf1*.

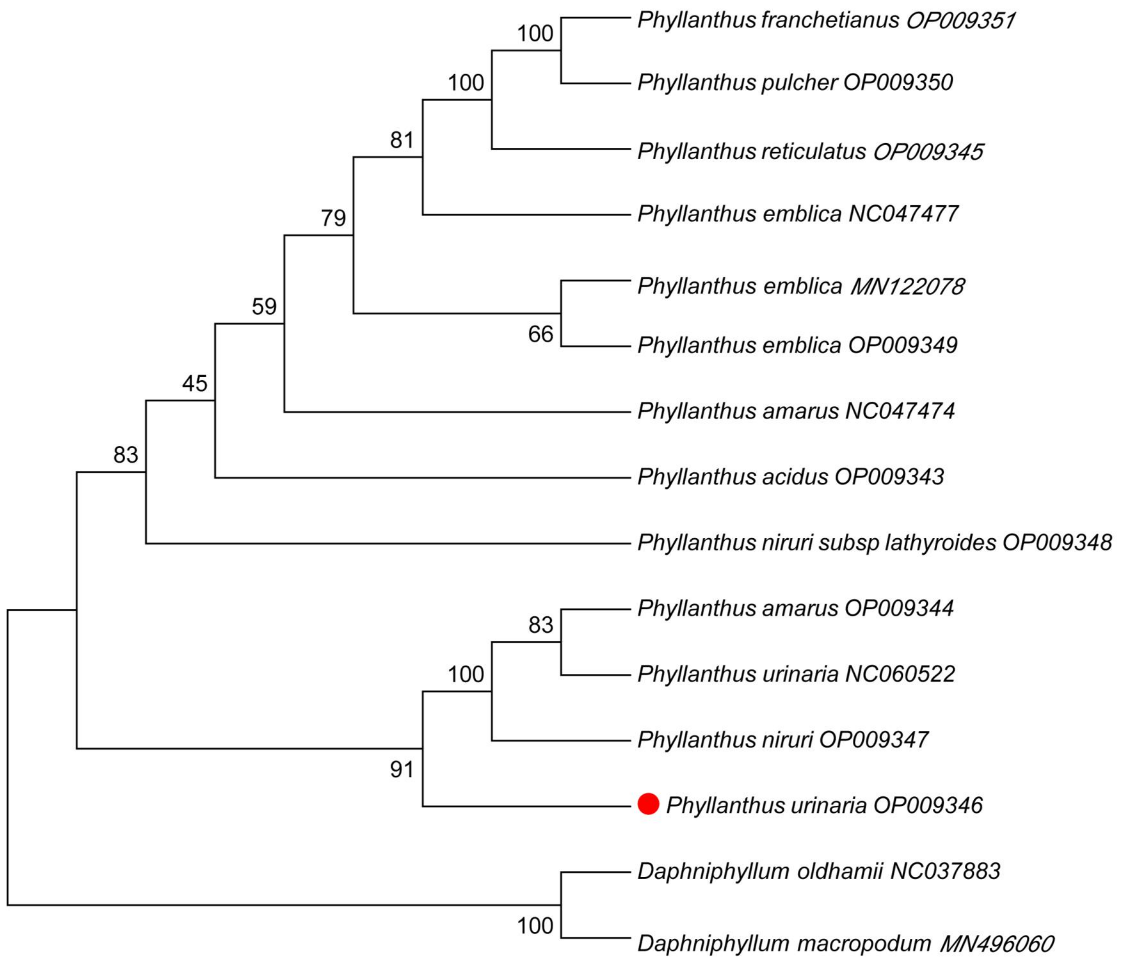

Fig. S4.10. Phylogenetic tree constructed using ML method based on *petA-psbJ.*

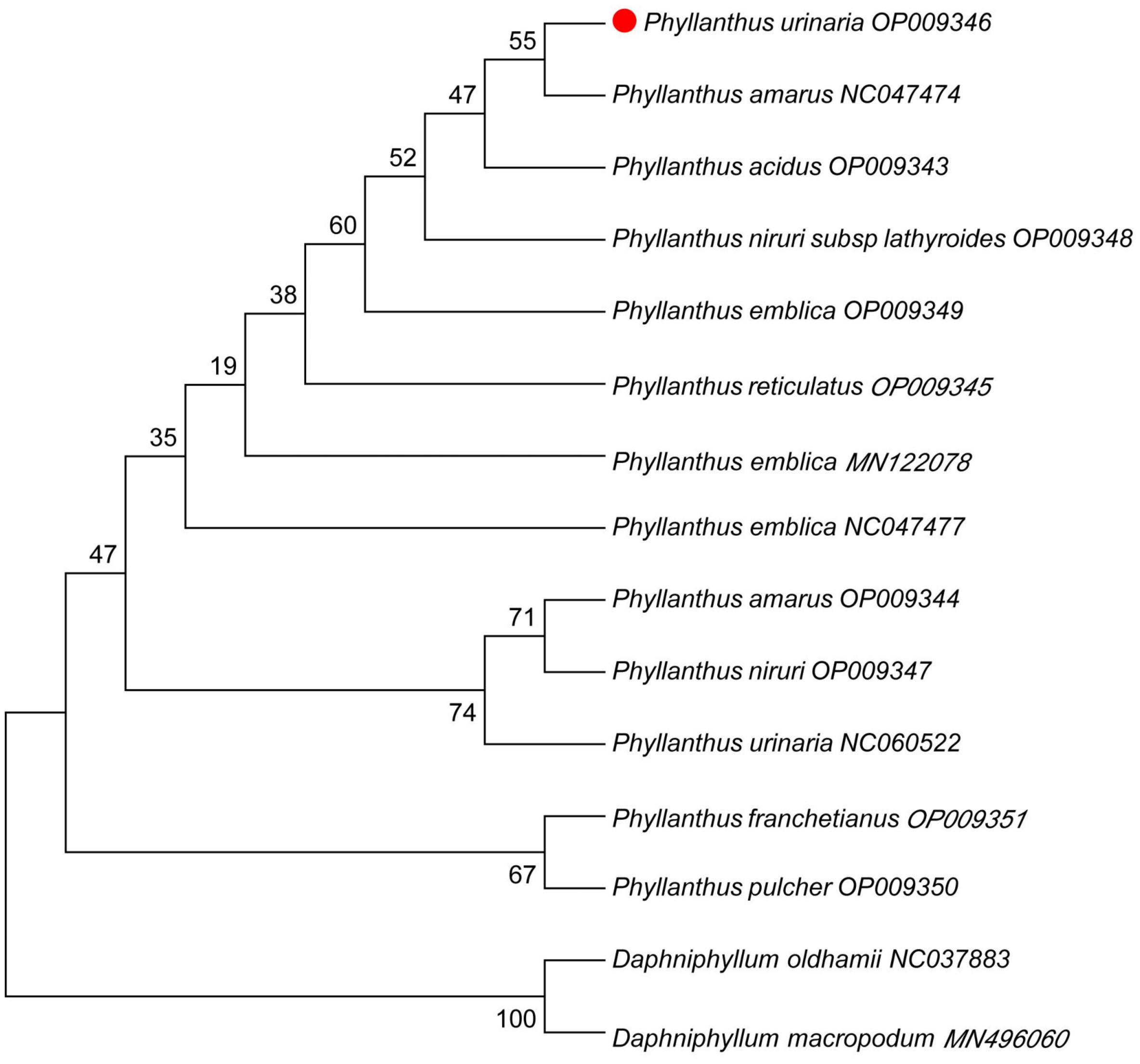

Fig. S4.11. Phylogenetic tree constructed using ML method based on *rrn5S-trnR-ACG*.

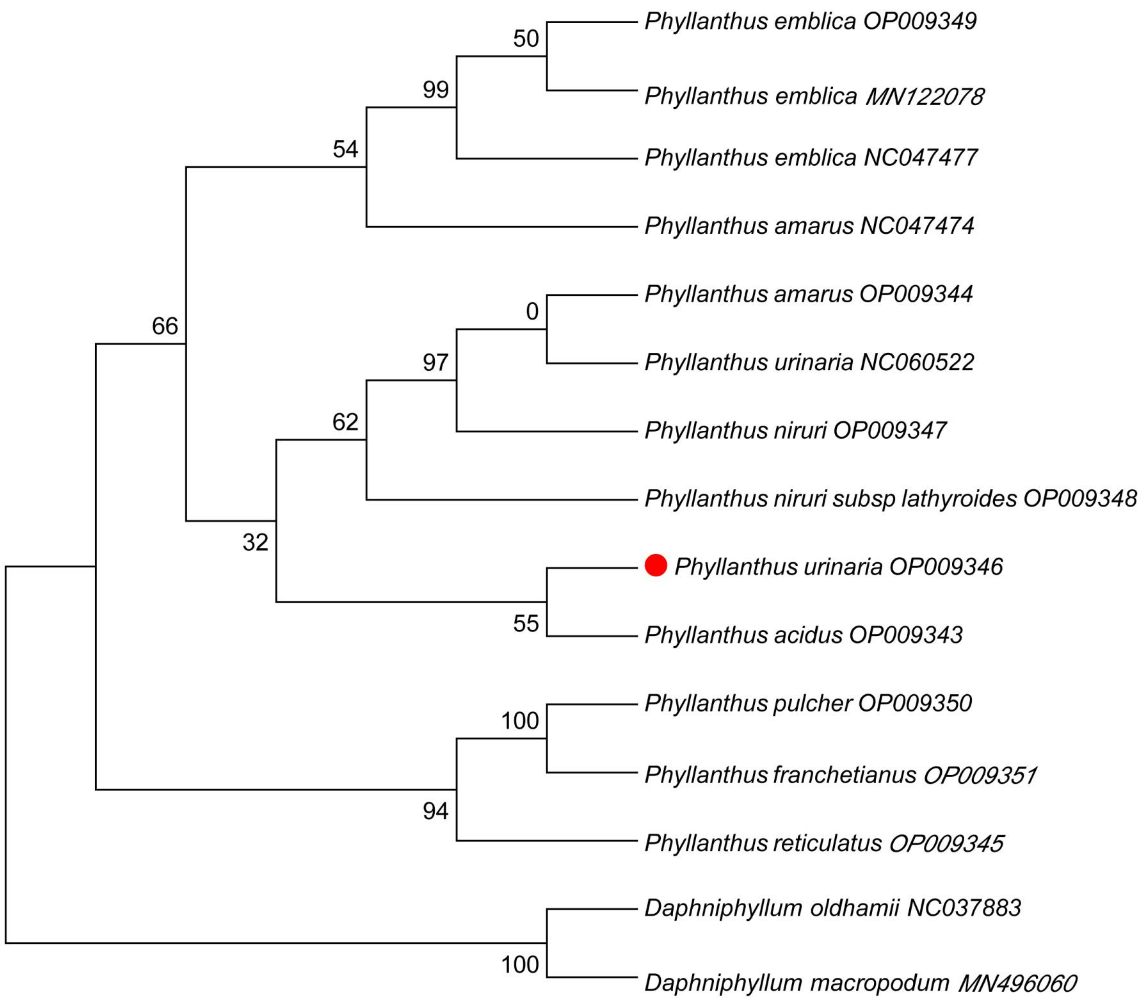

Fig. S4.12. Phylogenetic tree constructed using ML method based on *ndhE-ndhG*.

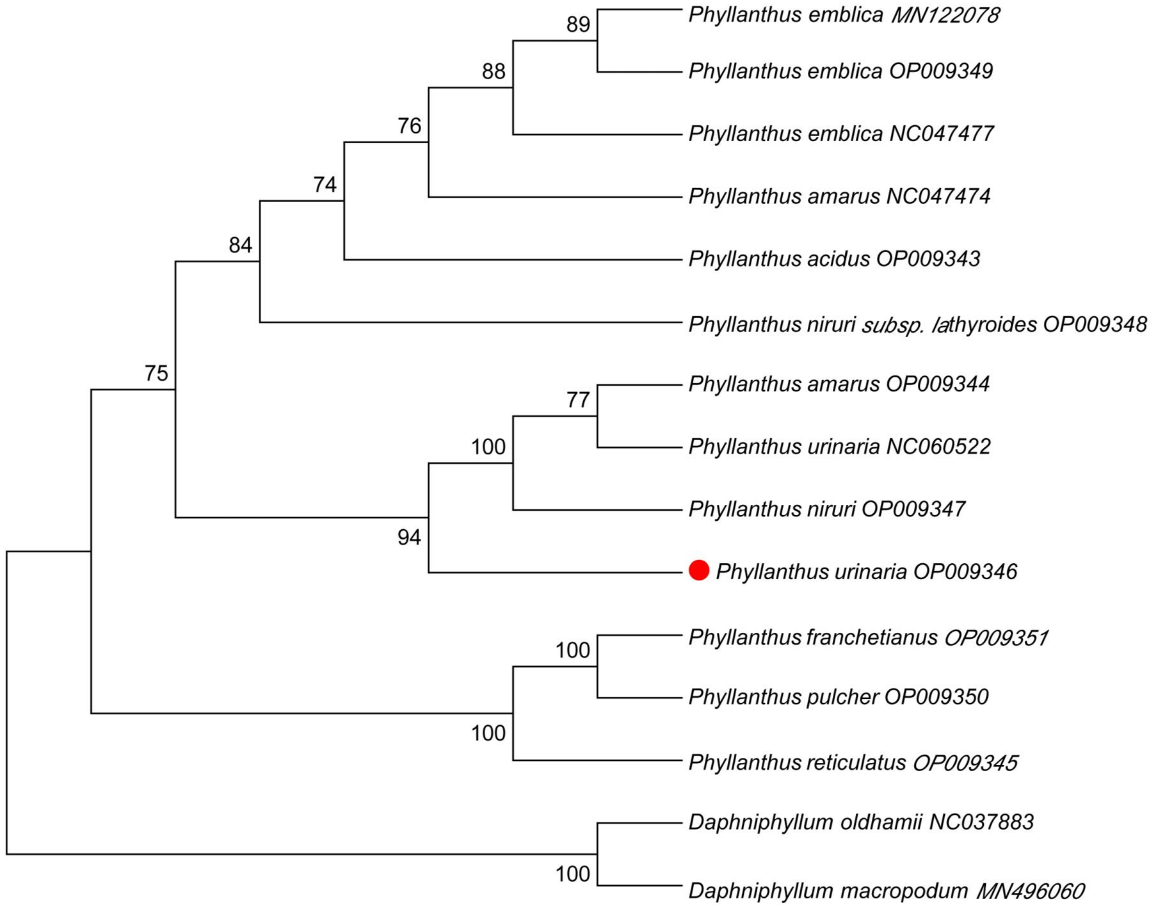

Fig. S5. Phylogenetic tree constructed using ML method based on the combination of three IGS.

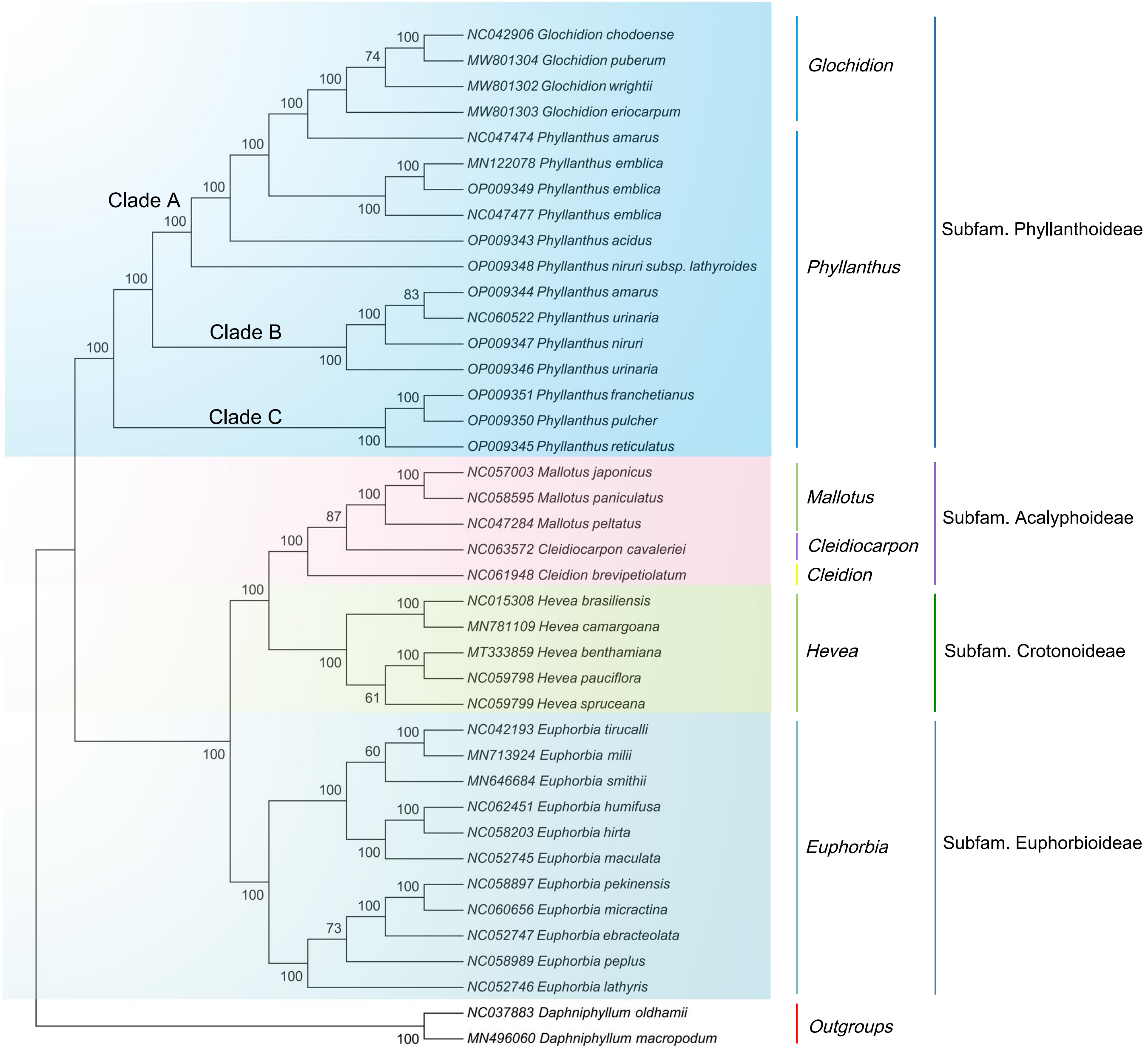

Fig. S6. NJ phylogenetic tree based on complete cp genomes. Numbers at nodes are bootstrap support values.

Table S1 The sample information of this study

No.	Species	Locality	Voucher specimen
1	*P. acidus*	Xishuangbannan, Yunnan, China	YXZ20220701
2	*P. amarus*	Xishuangbannan, Yunnan, China	YXZ20220702
3	*P. reticulatus*	Xishuangbannan, Yunnan, China	YXZ20220703
4	*P. urinaria*	Xishuangbannan, Yunnan, China	YXZ20220704
5	*P. niruri*	Dali, Yunnan, China	YXZ20220705
6	*P. niruri* subsp. *lathyroides*	Dali, Yunnan, China	YXZ20220706
7	*P. emblica*	Dali, Yunnan, China	YXZ20220707
8	*P. pulcher*	Dali, Yunnan, China	YXZ20220708
9	*P. franchetianus*	Dali, Yunnan, China	YXZ20220709

Table S2 Species information downloaded by NCBI

No.	Species	GenBank accession	No.	Species	GenBank accession
1	*Phyllanthus emblica*	NC047477	17	*Glochidion puberus*	MW801304
2	*Phyllanthus amarus*	NC047474	18	*Glochidion chodoense*	NC042906
3	*Phyllanthus urinaria*	NC060522	19	*Euphorbia ebracteolata*	NC052747
4	*Phyllanthus emblica*	MN122078	20	*Euphorbia hirta*	NC058203
5	*Cleidiocarpon cavaleriei*	NC063572	21	*Euphorbia humifusa*	NC062451
6	*Cleidion brevipetiolatum*	NC061948	22	*Euphorbia lathyris*	NC052746
7	*Mallotus japonicus*	NC057003	23	*Euphorbia maculata*	NC052745
8	*Mallotus paniculatus*	NC058595	24	*Euphorbia micractina*	NC060656
9	*Mallotus peltatus*	NC047284	25	*Euphorbia milii*	MN713924
10	*Hevea benthamiana*	MT333859	26	*Euphorbia pekinensis*	NC058897
11	*Hevea brasiliensis*	NC015308	27	*Euphorbia peplus*	NC058989
12	*Hevea camargoana*	MN781109	28	*Euphorbia smithii*	MN646684
13	*Hevea pauciflora*	NC059798	29	*Euphorbia tirucalli*	NC042193
14	*Hevea spruceana*	NC059799	30	*Daphniphyllum oldhamii*	NC037883
15	*Glochidion wrightii*	MW801302	31	*Daphniphyllum macropodum*	MN496060
16	*Glochidion eriocarpus*	MW801303			

Table S3 Genes in the *Phyllanthus* cp genomes.

Gene category	Gene group	Gene name
rRNA	rRNA genes	*rrn16S* (×2), *rrn23S* (×2), *rrn5S* (×2), *rrn4.5S* (×2)
tRNA	tRNA genes	*trnP-UGG*, *trnW-CCA*, *trnM-CAU*, **trnV-UAC*, *trnF-GAA*, **trnL-UAA*, *trnT-UGU*, *trnS-GGA*, *trnG-GCC*, *trnS-UGA*, *trnT-GGU*, *trnE-UUC*, *trnY-GUA*, *trnD-GUC*, *trnC-GCA*, *trnR-UCU*, **trnG-UCC*, *trnS-GCU*, *trnQ-UUG*, **trnK-UUU*, *trnH-GUG*, *trnI-CAU* (×2), *trnL-UAG*, *trnV-GAC* (×2), **trnI-GAU* (×2), **trnA-UGC* (×2), *trnR-ACG*(×2), *trnN-GUU* (×2), *trnL-CAA* (×2)
Photosynthesis	Photosystem I	*psaA*, *psaB*, *psaC*, *psaI*, *psaJ*
	Photosystem II	*psbA*, *psbB*, *psbC*, *psbD*, *psbE*, *psbF*, *psbI*, *psbJ*, *psbK*, *psbL*, *psbM*, *psbN*, *psbT*, *psbZ*, **ycf3*
	NADH-dehydrogenase	**ndhA*, **ndhB* (×2), *ndhC*, *ndhD*, *ndhE*, *ndhF*, *ndhG*, *ndhH*, *ndhI*, *ndhJ*, *ndhK*
	Cytochrome b/f complex	*petA*, *petB*, *petD*, *petG*, *petL*, *petN*
	ATP synthase	*atpA*, *atpB*, *atpE*, *atpF*, *atpH*, *atpI*
	Rubisco	*rbcL*
Self replication	Large subunit of ribosome	*rpl14*, *rpl16*, **rpl2* (×2), *rpl20*, *rpl22*, *rpl23* (×2), *rpl33*, *rpl36*
	Small subunit of ribosome	*rps11*, *rps14*, *rps15*, **rps16*, *rps18*, *rps19*(×2), *rps3*, *rps4*, *rps7* (×2), *rps8*, *rps2*
	DNA dependent RNA polymerase	*rpoA*, *rpoB*, **rpoC1*, *rpoC2*
Other genes	Acetyl-CoA-carboxylase	*accD*
	c-type cytochrom synthesis gene	*ccsA*
	Envelop membrane protein	*cemA*
	Protease	**clpP*
	Maturase	*matK*
Unkown	Conserved open reading frames	*ycf1*, *ycf2* (×2), *ycf4*

Intron-containing genes are marked by asterisks (*).

Table S4 The simple sequence repeat (SSR) types of the nine cp genomes of *Phyllanthus* species

**SSR type**	**Repeat unit**	**Amount**								
		P. acidus	***P. amarus***	***P. reticulatus***	***P. urinaria***	***P. niruri***	***P. niruri* subsp. *lathyroides***	***P. emblica***	***P. pulcher***	***P. franchetianus***
**Mono**	**A/T**	33	64	36	48	63	28	54	40	40
	**C/G**	0	3	1	1	3	0	0	0	0
**Di**	**AC/GT**	0	0	0	1	0	0	0	0	0
	**AG/CT**	2	2	2	4	2	2	2	0	0
	**AT/AT**	10	18	14	13	18	17	14	15	15
**Tri**	**AAG/CTT**	3	2	2	2	2	3	3	0	0
	**AAT/ATT**	3	3	2	5	3	1	3	3	3
**Tetra**	**AAAG/CTTT**	1	1	2	1	1	1	1	2	2
	**AAAT/ATTT**	2	2	1	3	2	1	3	3	3
	**AATT/AATT**	2	2	1	1	2	1	1	3	3
	**AGAT/ATCT**	0	1	0	0	0	0	0	0	0
	**ACAG/CTGT**	0	0	0	0	1	0	0	1	1
	**ACGG/CCGT**	0	0	0	0	0	0	0	1	1
**Penta**	**AATAT/ATATT**	0	0	1	0	0	0	0	1	1
**Hexa**	**ACATAT/ATATGT**	0	0	0	1	0	0	0	0	0

Table S5 Codons in cp genome of nine *Phyllanthus* species.

Amino	Codon	RSCUa																			
		P.acidus	***P. amarus***	***P. reticulatus***	***P. urinaria***	***P. niruri***	***P. niruri* subsp. *lathyroides***	***P. emblica***	***P. pulcher***	***P. franchetianus***			***P.acidus***	***P. amarus***	***P. reticulatus***	***P. urinaria***	***P. niruri***	***P. niruri subsp. lathyroides***	***P. emblica***	***P. pulcher***	***P. franchetianus***
Ala	GCU	1.77	1.69	1.82	1.74	1.69	1.78	1.79	1.86	1.86	Leu	CUC	0.33	0.44	0.29	0.44	0.43	0.39	0.39	0.34	0.34
	GCC	0.64	0.67	0.61	0.66	0.67	0.65	0.67	0.62	0.62		CUA	0.84	0.88	0.83	0.87	0.88	0.83	0.72	0.71	0.71
	GCA	1.12	1.11	1.11	1.07	1.11	1.06	1.04	1.01	1.02		CUG	0.4	0.47	0.37	0.46	0.47	0.43	0.37	0.4	0.4
	GCG	0.48	0.53	0.47	0.53	0.53	0.5	0.5	0.51	0.5	Lys	AAA	1.51	1.41	1.59	1.42	1.4	1.45	1.53	1.54	1.55
Arg	CGU	1.36	1.09	1.37	1.13	1.09	1.22	0.95	1.08	1.06		AAG	0.49	0.59	0.41	0.58	0.6	0.55	0.47	0.46	0.45
	CGC	0.41	0.38	0.41	0.41	0.38	0.39	0.4	0.45	0.44	Met	AUG	1	1	1	1	1	1	1	1	1
	CGA	1.37	1.25	1.47	1.25	1.25	1.26	1.17	1.24	1.23	Phe	UUU	1.34	1.27	1.38	1.27	1.27	1.32	1.37	1.4	1.4
	CGG	0.42	0.63	0.43	0.55	0.63	0.58	0.45	0.51	0.5		UUC	0.66	0.73	0.62	0.73	0.73	0.68	0.63	0.6	0.6
	AGA	1.79	1.87	1.8	1.87	1.88	1.74	2.08	1.82	1.86	Pro	CCU	1.59	1.48	1.68	1.51	1.47	1.48	1.52	1.56	1.55
	AGG	0.66	0.78	0.52	0.79	0.77	0.8	0.96	0.9	0.91		CCC	0.71	0.74	0.68	0.75	0.74	0.72	0.7	0.86	0.86
Asn	AAU	1.51	1.45	1.53	1.44	1.45	1.47	1.45	1.45	1.46		CCA	1.1	1.08	1.06	1.04	1.08	1.07	1.1	1	1.01
	AAC	0.49	0.55	0.47	0.56	0.55	0.53	0.55	0.55	0.54		CCG	0.6	0.7	0.58	0.7	0.71	0.73	0.68	0.58	0.58
Asp	GAU	1.56	1.51	1.57	1.52	1.51	1.49	1.56	1.57	1.57	Ser	UCU	1.73	1.52	1.76	1.43	1.52	1.59	1.58	1.57	1.57
	GAC	0.44	0.49	0.43	0.48	0.49	0.51	0.44	0.43	0.43		UCC	0.93	0.91	0.87	0.94	0.91	0.89	0.85	0.84	0.83
Cys	UGU	1.46	1.34	1.52	1.33	1.34	1.34	1.23	1.27	1.26		UCA	1.22	1.33	1.2	1.28	1.33	1.26	1.25	1.13	1.15
	UGC	0.54	0.66	0.48	0.67	0.66	0.66	0.77	0.73	0.74		UCG	0.54	0.7	0.49	0.76	0.7	0.69	0.63	0.6	0.62
Gln	CAA	1.55	1.44	1.54	1.41	1.44	1.43	1.56	1.55	1.56		AGU	1.23	1.06	1.35	1.12	1.06	1.09	1.04	1.18	1.16
	CAG	0.45	0.56	0.46	0.59	0.56	0.57	0.44	0.45	0.44		AGC	0.36	0.47	0.34	0.48	0.47	0.48	0.66	0.67	0.67
Glu	GAA	1.51	1.48	1.54	1.5	1.48	1.49	1.51	1.53	1.54	Thr	ACU	1.53	1.37	1.66	1.22	1.37	1.43	1.39	1.54	1.54
	GAG	0.49	0.52	0.46	0.5	0.52	0.51	0.49	0.47	0.46		ACC	0.76	0.78	0.73	0.95	0.78	0.78	0.89	0.86	0.86
Gly	GGU	1.34	1.29	1.38	1.27	1.29	1.31	1.17	1.29	1.29		ACA	1.28	1.25	1.23	0.83	1.25	1.19	1.16	1.14	1.13
	GGC	0.44	0.51	0.45	0.51	0.51	0.52	0.54	0.53	0.52		ACG	0.43	0.6	0.38	1.36	0.6	0.6	0.57	0.46	0.47
	GGA	1.56	1.42	1.56	1.41	1.42	1.44	1.48	1.44	1.44	Trp	UGG	1	1	1	0.76	1	1	1	1	1
	GGG	0.66	0.79	0.62	0.81	0.79	0.73	0.81	0.75	0.75	Tyr	UAU	1.57	1.48	1.63	1.24	1.48	1.49	1.51	1.54	1.54
His	CAU	1.46	1.32	1.52	1.32	1.32	1.35	1.46	1.47	1.47		UAC	0.43	0.52	0.37	0.63	0.52	0.51	0.49	0.46	0.46
	CAC	0.54	0.68	0.48	0.68	0.68	0.65	0.54	0.53	0.53	Val	GUU	1.49	1.39	1.49	1	1.39	1.34	1.56	1.53	1.55
Ile	AUU	1.51	1.37	1.52	1.39	1.37	1.46	1.62	1.62	1.61		GUC	0.48	0.56	0.43	1.47	0.57	0.55	0.57	0.46	0.46
	AUC	0.57	0.71	0.53	0.69	0.71	0.62	0.7	0.64	0.64		GUA	1.47	1.4	1.54	0.53	1.4	1.46	1.33	1.44	1.43
	AUA	0.92	0.92	0.94	0.92	0.92	0.91	0.69	0.75	0.75		GUG	0.56	0.64	0.54	1.39	0.64	0.65	0.55	0.57	0.56
Leu	UUA	2.02	1.72	2.13	1.71	1.72	1.8	1.95	2.07	2.07	Stop	UAA	1.28	1.2	1.48	0.54	1.21	1.3	1.17	1.35	1.36
	UUG	1.16	1.31	1.13	1.31	1.31	1.34	1.26	1.24	1.26		UAG	0.91	0.94	1.04	1.41	0.93	0.96	0.6	0.61	0.61
	CUU	1.26	1.19	1.26	1.21	1.19	1.21	1.31	1.24	1.22		UGA	0.81	0.86	0.48	0.66	0.86	0.74	1.23	1.04	1.04
